# Modification of the Mineral Quality of Wheat After the Application of Selenium and Sulfur

**DOI:** 10.3390/molecules31081283

**Published:** 2026-04-14

**Authors:** Marzena S. Brodowska, Magdalena Kurzyna-Szklarek, Mirosław Wyszkowski

**Affiliations:** 1 Department of Agricultural and Environmental Chemistry, University of Life Sciences in Lublin, Akademicka 15 Str., 20-950 Lublin, Poland; marzena.brodowska@up.lublin.pl (M.S.B.);; 2 Department of Agricultural and Environmental Chemistry, University of Warmia and Mazury in Olsztyn, Łódzki 4 Sq., 10-727 Olsztyn, Poland

**Keywords:** selenium, sulfur, spelt wheat, common wheat, macroelements

## Abstract

The mineral composition of cereals is one of the key indicators of the quality of agricultural raw materials, determining both nutritional value and technological and processing properties. Complex interactions between nutrients, especially sulfur and selenium, can significantly modify the accumulation of macroelements in plant tissues. The aim of the study was to assess the effect of different doses of sulfur (S_1_—15 kg S ha^−1^ and S_2_—30 kg S ha^−1^) and selenium (Se_1_—10 g Se ha^−1^ and Se_2_—20 g Se ha^−1^), as well as the timing of selenium application, on the phosphorus, potassium, calcium and magnesium contents in the grain and straw of spelt and common wheat. The results obtained indicate clear interspecies differences and a non-linear, often species-specific response to selenium doses. In common wheat grain, the application of selenium at two doses increased potassium and magnesium contents by 4–9% and 4–11%, respectively, and it reduced calcium content by 14–18% in spelt wheat grain. In spelt wheat straw, selenium application resulted in an 11% decrease in potassium content and an 8–10% decrease in calcium content. In common wheat, on the other hand, the straw responded with a 17% (Se_1_) and 13% (Se_2_) increase in magnesium content, accompanied by an 8–10% decrease in potassium content. Sulfur exhibited species-specific effects. In spelt wheat straw, it increased phosphorus content by 5–10%, calcium by 11% and magnesium by 15%. In common wheat straw, sulfur also reduced potassium accumulation by 5% and calcium by 23% (S_1_) and 9% (S_2_). The timing of selenium application modified the results of their content, but did not show a universal reaction pattern: earlier application increased the P content in spelt straw, while later application promoted an increase in Ca content in common wheat grain.

## 1. Introduction

Macronutrients are the basic group of minerals that determine the growth, development and yield of plants [1,2]. Key structural and regulatory functions are performed by elements such as nitrogen, phosphorus, potassium, calcium, magnesium and sulfur, which participate in the synthesis of proteins, nucleic acids, chlorophyll and enzymes, as well as maintaining the ionic and osmotic balance of plants [3,4]. The availability of these elements in the soil and their proportions are essential to ensure a high rate of photosynthesis, proper root system growth, efficient transport of assimilates and effective use of water. These factors directly impact the yield and quality of wheat [5].

Phosphorus is a very important macroelement for wheat. It stimulates the development of the root system, enabling the plant to absorb other nutrients more efficiently, access water more easily and withstand drought stress more effectively [6,7]. For this reason, the beginning of plant development is the period of greatest phosphorus demand, so for maximum effectiveness, it is best to apply P before sowing. Phosphorus also influences the proper branching of plants and increases their resistance to frost and water shortage. An optimal supply of phosphorus prevents wheat lodging, increases protection against fungal diseases and shortens grain ripening time [6,8]. Nitrogen fertilisation and soil pH significantly influence phosphorus uptake by plants. In alkaline soils, phosphorus forms poorly soluble compounds with calcium and magnesium, whereas in acidic soils, iron and aluminium phosphate are formed, which are forms unavailable to plants [9,10]. This macroelement P is exclusively absorbed by plants in ionic form, H_2_PO_4_^−^ and HPO_4_^2−^, which diffuse deep into the root system [7,11].

Plants absorb the largest amounts of potassium during the period from stem elongation to flowering, as it is mainly responsible for regulating water management. Therefore, it should be present in the soil throughout the plant’s development [12]. Due to the fact that potassium is an element that easily moves in the soil, fertilisation with K requires consideration of soil conditions. The movement of potassium in the soil is influenced by low organic-matter content, high pH, limited mineral fertilisation and high clay-mineral content [13]. Drought can also result in potassium deficiency. It manifests as chlorosis of the leaf tips and edges, and in the later stages as necrosis [14]. On the other hand, excessive potassium fertilisation can result in soil salinisation [15]. Potassium in the soil occurs in forms of varying availability, mainly bound to clay minerals such as illite or micas. Clay minerals can immobilise K in interlayer spaces, limiting its availability to plants [13]. The mobility of potassium depends on the mineral and organic properties of the soil. Humic substances can stabilise K or, in the case of fulvic acids, increase its migration in the soil profile [16]. Soil pH significantly affects K sorption. In alkaline soils, its binding by Ca^2+^ and Mg^2+^ increases, promoting retrogression. Lower pH increases mobility but also susceptibility to leaching [17]. Under drought conditions, limited ion diffusion reduces potassium availability, and K supplementation improves the ability of plants to regulate water management and metabolic responses [18]. Organic matter also plays an important role. Humic acids increase potassium retention, whereas fulvic acids increase its availability and the efficiency with which potassium is used in fertilisers; this has been documented by an increase in NPK utilisation of over 20% [19].

Calcium is a component of cell walls, affecting their permeability and stabilising the structure of pectins. It also strengthens the bonds between molecules within the apoplast. In conditions of water shortage, calcium stabilises micelles and cell membranes, preventing their damage and loss of integrity [20]. The only form of calcium available to plants is the ionic form Ca^2+^. It is absorbed by the roots and transported mainly as a result of transpiration in the xylem [21]. Calcium also acts as a secondary signalling messenger, participating in plant responses to environmental stress, including drought, salinity and low temperatures. In these situations, specific ”calcium signatures” activate stress-response networks [22]. Soil acidification promotes the release of toxic aluminium (Al^3+^) ions, which inhibit the development of the wheat root system and disrupt nutrient uptake [23]. Applying lime to the soil increases the pH of the soil solution, reducing aluminium toxicity and improving the availability of calcium and magnesium. Additionally, it alters the levels of manganese, zinc, and other trace elements in the grain, affecting their bioavailability and the structure of the sorption complex [24]. Calcium, as a key regulator of membrane integrity and the cell skeleton, also increases plant resistance to abiotic stresses (drought and high temperatures) and biotic stresses (pathogens). This is partly due to its role in stabilising membrane phospholipids and regulating ion transport [25]. Calcium, as an important basic cation, influences magnesium metabolism by regulating cation ratios in the soil and competition for sorption sites; its presence affects the stability of cell membranes, cell walls, and stress signalling. In addition, under pH-regulation conditions (e.g., after liming), calcium supports Mg^2+^ uptake by plants and reduces aluminium toxicity, thereby improving root system growth [20,22]. Recent studies on calcium signalling in the regulation of plant mineral nutrition have confirmed that calcium, as a secondary signal, also influences the regulation of Mg^2+^ transporters and their activation in response to environmental stress [22].

Magnesium is an essential element for building chlorophyll as it constitutes the central ion in the chlorophyll molecule. This is why it is mainly found in chloroplasts and is necessary for photosynthesis [26]. Magnesium acts as a cofactor for numerous enzymes involved in the metabolism of proteins, carbohydrates and fats, stabilises ribosomes and participates in ATP-related reactions, increasing the nutrient content of the grain. Proper magnesium nutrition also increases the resistance of wheat to short-term water deficiency and low temperatures, which is related to the role of Mg in maintaining membrane integrity and photosynthetic efficiency [27]. Magnesium is absorbed in the cationic form Mg^2+^ by both roots and leaves, and its homeostasis is regulated by specialised MGT/MRS2 transporters that are responsible for the uptake, distribution and storage of Mg^2+^ in plants [26]. Soil pH and soil nitrogen and phosphorus supply have a significant impact on magnesium uptake by plants. The optimal pH range for its availability is between 5.5 and 7.2; however, low organic-matter content, acidification and competition from other cations (Ca^2+^, K^+^) can limit its uptake [26]. Deficiency of this macroelement often remains hidden, but it leads to a slowdown in the development of winter wheat, limits the uptake of other nutrients—especially phosphorus—reduces plant resistance to fungal diseases, decreases the amount of protein in the grain and impairs crop quality. Most importantly, it disrupts the synthesis of chlorophyll, which is key in the process of photosynthesis [26,27].

In the soil environment, calcium, magnesium and potassium interact strongly due to their joint participation in cation-exchange sorption. Changes in the availability of one of these elements affect the uptake of the others. Calcium, by stabilising the structure of cell membranes and regulating ion transport, also modifies the selectivity of channels and transporters responsible for the uptake of Mg^2+^ and K^+^, which results from its role in mineral signalling and stress responses [22]. Under conditions of soil acidification, the concentration of toxic Al^3+^ increases, which disrupts the uptake of all basic cations, including Ca^2+^, Mg^2+^ and K^+^. Only liming can raise the pH, thereby reducing the activity of aluminium and improving the availability of basic cations, which has been confirmed in studies on pH and its impact on the availability of calcium, magnesium and potassium [23]. As a result, the Ca–Mg–K balance becomes crucial for maintaining proper cell-membrane function, water management and enzymatic activity, especially under environmental stress conditions [24].

Ca^2+^, as the cation with the strongest binding affinity, may limit the excessive mobility of K^+^ and Mg^2+^ in light soils, thereby promoting their more balanced uptake by plants. Micronutrients are essential for the proper growth of winter wheat as they participate in key metabolic processes as enzyme components or activators [28]. Elements such as Mn, Fe, Zn, Cu, B, and Mo regulate numerous metabolic pathways, including photosynthesis, cellular respiration, redox processes, hormone synthesis, and plant defence mechanisms; for example, Mn participates in water photolysis, Fe in chlorophyll synthesis and electron transport, Zn activates dehydrogenases and participates in auxin synthesis, Cu performs redox functions, and Mo is a component of nitrogen assimilation enzymes [28,29].

Selenium and sulfur were selected for the study due to their exceptionally strong biochemical links and the fact that the anions co-occur in the soil; in addition, sulfates and selenates compete for the same transport systems in plant roots [30,31]. Selenium uptake occurs mainly via sulfate transporters, and its metabolism follows the same assimilation pathway as that of sulfur, involving shared enzymes such as ATP sulfurylase and APS reductase [32]. An excessive supply of sulfur limits selenium uptake due to antagonism at the transporter level, whilst a deficiency may increase Se accumulation, though often at the expense of biomass growth [33]. This close relationship between the availability of S and Se means that understanding their mutual interactions is crucial for developing effective biofortification strategies and minimising the risk of toxicity at high selenium doses [30,31].

The importance of selenium as a deficient element in the human and animal diet further justifies the need for research into its uptake and utilisation by plants. In many countries, such as the United Kingdom and the Nordic countries, the natural selenium content in wheat is low (7–22 μg·kg^−1^ dry matter), whilst values considered nutritionally adequate are several times higher [34,35]. Optimal doses of selenium fertilisers are relatively low (4–13 g·ha^−1^), but their effectiveness depends heavily on interactions with macronutrients—particularly sulfur—which can reduce Se uptake by tens of percent [36]. Recent studies confirm that sulfur fertilisation strategies significantly modulate the effectiveness of selenium biofortification—excess S reduces Se accumulation in grain, whilst a deficiency increases uptake but may limit plant growth [37]. Therefore, a simultaneous analysis of the roles of S and Se is essential for developing sustainable fertilisation methods that will enhance the nutritional value of crops whilst maintaining their high quality. The sulfur content of the soil strongly influences the availability and uptake of other macronutrients, such as phosphorus, potassium, calcium and magnesium, as S modifies both the soil redox potential and the activity of the microorganisms responsible for nutrient release [38]. Increased sulfur mineralisation often enhances the mobility of P and K, whilst in the case of Ca and Mg, it may influence their movement within the soil profile by altering pH and local ionic interactions [39]. The combined application of sulfur and selenium further modifies the dynamics of these elements, as S and Se compete for similar transport pathways within the plant, which may alter the uptake of P, Mg and Ca depending on their proportions in the soil and the chemical form of the applied fertilisers [40]. Selenium, particularly in its more mobile forms, can alter the composition of the rhizosphere microbiome, indirectly influencing the cycling of P and K and the availability of sulfates in the root zone [41]. Consequently, both Se and S act as regulators of interactions between macronutrients, and their combined application may lead to synergy or antagonism, depending on site conditions and fertiliser form, which is crucial for optimising fertilisation in soil–plant systems.

It is essential to determine the nutrient content of wheat grain and straw separately, as these two parts of the plant perform different physiological functions and differ in their ability to accumulate nutrients. The grain is the primary source of nutrients for humans and animals, so its composition determines the nutritional value of the crop, whilst the straw is the main store of many bioelements and reflects the plant’s response to fertilisation and growing conditions. Analysing both fractions separately allows for a precise assessment of the effectiveness of biofortification, the pathways of nutrient transport within the plant, and potential losses or accumulation in organs other than the marketable crop, which is essential for optimising fertilisation strategies and improving food quality [42].

Macronutrients such as N, P, K, Ca, Mg and S are crucial for wheat growth and yield as they regulate metabolic processes, tissue development and plant water management [1,2,3,4]. Phosphorus supports root system development, increases plant stress tolerance and influences proper tillering; however, its availability is strongly dependent on soil pH and its chemical form in the soil solution [6,7,8,9,10,11]. Potassium plays a significant role in regulating water management, and its availability depends on soil type, organic-matter content, pH and moisture conditions, with both a deficiency and an excess of K limiting plant growth [13,14,15,16,17,18,19]. The interactions between Ca, Mg and K in the sorption complex and the role of Ca^2+^ as a regulator of ion transport influence nutrient uptake, particularly under stress conditions and in the presence of toxic Al^3+^ [20,21,22,23,24,25]. Sulfur and selenium, which compete for shared transport systems, significantly modify the availability of macro- and micronutrients, and their ratios in the soil determine the effectiveness of biofortification and the accumulation of elements in grain and straw, which are crucial for assessing nutritional value and the effectiveness of fertilisation [30,31,32,33,34,35,36,37,38,39,40,41,42].

The mineral composition of cereals is a key parameter determining the quality of agricultural raw materials, as it influences both their nutritional value and technological and processing properties. Interactions between mineral elements, in particular sulfur and selenium, can significantly affect the accumulation of macroelements in plant tissues. The aim of this study was to evaluate the effects of different doses of sulfur and selenium, as well as the timing of selenium application, on the phosphorus, potassium, calcium and magnesium contents in the grain and straw of spelt wheat and common wheat.

## 2. Results and Discussion

### 2.1. Phosphorus in Wheat

The phosphorus content in both grain and straw (Table 1 and Table 2) in both test plants was consistent with the values most commonly found in the literature [32].

The application of selenium was associated with a slight decrease in phosphorus content in spelt and common wheat grain. The timing of selenium application did not clearly differentiate the phosphorus content in spelt and common wheat grain. Fertilisation with the first dose of sulfur was associated with a slight decrease in phosphorus content in spelt and common wheat grain.

Selenium fertilisation was associated with an 8% reduction in phosphorus content in spelt wheat straw, regardless of the dose applied. The application of a lower dose of selenium resulted in an 11% increase in phosphorus content in common wheat straw, while the application of a higher dose resulted in the opposite reaction—a 5% decrease in phosphorus content. However, the timing of selenium application had an impact on the increase in phosphorus content. Higher phosphorus content in spelt wheat straw was recorded in facilities where selenium was applied earlier. The timing of selenium application did not clearly differentiate the phosphorus content in common wheat straw. Sulfur fertilisation resulted in an increase in phosphorus content in spelt wheat straw, with a 5% lower dose and a 10% higher dose. Sulfur fertilisation, in most cases, was associated with an increase in phosphorus content in common wheat straw, by up to 9% in the case of fertilisation with a lower dose of this component.

Selenium fertilisation resulted in a reduction in phosphorus content in winter wheat, which is attributed to competition between selenate (SeO_4_^2−^) and phosphate (PO_4_^3−^) ions for common anion transporters and changes in subcellular phosphorus distribution under the influence of selenium availability [43]. Studies on wheat grown in a hydroponic system revealed that higher doses of phosphorus, when applied simultaneously with selenium, reduced Se uptake and altered its transport. This led to a decrease in phosphorus concentration in plant organs due to changes in the ratio of phosphorus fractions and interactions between P and Se metabolism [44].

In turn, studies on sulfur fertilisation in spring and winter wheat report no clear effect of sulfur on phosphorus content in grain, which was explained by the stability of phosphate transport under conditions of increased sulfur supply and the priority use of S in the synthesis of sulfur amino acids [45]. However, Liu et al. [44] found that increased sulfur fertilisation can lead to a slight reduction in phosphorus content in the above-ground parts of wheat. This mechanism was associated with competition between sulfates (SO_4_^2−^) and phosphates for common transport pathways and their different effects on the activity of enzymes responsible for anion metabolism in the plant. Hydroponic studies have also shown that even with varying nitrogen fertilisation, increased sulfur supply can reduce phosphorus availability in wheat by shifting phosphorus to less mobile fractions and increasing the activity of sulfur metabolism processes [44].

### 2.2. Potassium in Wheat

The potassium content in wheat (Table 3 and Table 4) was similar to the data in the literature [46]. The presence of selenium in the spelt-wheat growing environment and the timing of its application were not associated with significant changes in the potassium content of its grain. The application of selenium at a lower dose caused a slight increase in potassium content by 4%, and at a higher dose by 9% in common wheat grain. The timing of selenium application did not have a clear effect on the potassium content in common wheat grain. A similar lack of correlation in spelt wheat grain was observed in the case of sulfur fertilisation. The presence of both lower and higher amounts of sulfur in the plant growth environment, in most cases, was associated with a slightly lower potassium content in common wheat grain. The application of a lower dose of sulfur resulted in a 4% decrease in potassium content.

In spelt wheat straw, selenium fertilisation in most facilities resulted in a reduction in potassium content, and the application of a lower dose was associated with an average reduction in its accumulation of 11%. The addition of selenium caused a reduction in potassium content in common wheat straw of 10% and 8%, respectively. However, the timing of selenium application did not cause any clear changes in the potassium concentration in spelt wheat and common wheat straw. The addition of sulfur to the spelt-wheat growing environment resulted in a reduction in potassium content in its straw in most cases. The application of a lower dose of sulfur resulted in a 6% lower potassium content compared to cases without sulfur fertilisation. Fertilisation with both higher and lower doses of sulfur, in most cases, was associated with a decrease in the potassium content of common wheat straw. The application of a lower dose resulted in a 5% decrease in potassium content.

Similar observations regarding the reduction in potassium content in wheat grain under the influence of selenium fertilisation were reported by Ducsay et al. [47]. They showed that the use of sodium selenite led to a decrease in potassium levels, which was explained by disturbances in ion metabolism resulting from competition between SeO_3_^2−^ ions and K^+^ cations and the potential influence of Se on the activity of membrane proton pumps that regulate cation uptake. Studies on wheat seedlings exposed to stress factors have also shown that the presence of selenium can modify potassium transport. Studies by Zembala et al. [48] showed that Se, especially under conditions of additional stress (e.g., cadmium excess), reduced K uptake by plants and decreased its effective distribution in tissues. This is associated with damage to cell membranes and disruption of the electrochemical gradients responsible for ion transport. The mechanisms of these relationships include: competition between selenium anions (SeO_3_^2−^/SeO_4_^2−^) and other elements for common transporters; the effect of selenium on cell-membrane integrity, which alters cation permeability; and modulation of H^+^-ATPase and potassium channels, which affects K uptake and relocation. The action of selenium as a stress factor at higher doses can reduce K content in grain through increased oxidative damage and osmoregulation disorders. In summary, available studies on wheat confirm that selenium can reduce potassium content, especially in grain. The strength of this effect depends on the form of Se, the dose and the physiological condition of the plants.

### 2.3. Calcium in Wheat

The average calcium content in spelt wheat grain was 1.49 g kg^−1^ dry matter, and in common wheat grain it was 1.69 g kg^−1^ dry matter (Table 5). In wheat straw (Table 6), these values were 6.57 and 6.69 g kg^−1^ dry matter, respectively. These values are consistent with the literature data [49]. Selenium fertilisation was associated with a decrease in calcium content in spelt wheat grain by an average of 14 and 18%. The timing of selenium application did not result in a clear change in calcium content in spelt wheat grain. Common wheat responded with a decrease in calcium content after a lower dose of selenium was applied, while a 10% increase in calcium content was observed in plots fertilised with a higher dose. On average, calcium content in common wheat grain was 11% higher in plots fertilised with selenium at a later date. The application of a lower dose of sulfur was associated with a 7% decrease in calcium content in spelt wheat grain and a 5% decrease in common wheat grain.

The application of selenium resulted in a reduction in calcium content in spelt wheat straw by 10% and 8%, respectively. In common wheat straw, fertilisation with a lower dose of selenium was associated with a 10% increase in calcium content, while a higher dose resulted in an 8% decrease. Later fertilisation with this element resulted in an average 7% decrease in calcium content in spelt wheat straw. The timing of selenium application did not have a clear effect on the calcium content in common wheat straw. Fertilisation with a higher dose of sulfur resulted in an 11% increase in calcium content in spelt wheat straw. The presence of sulfur in the plant growth environment caused a decrease in calcium content in common wheat straw. The application of a lower dose of S resulted in a decrease in its accumulation in common wheat straw by an average of 23%, and a higher dose by 9%.

The literature contains information on both the positive and negative effects of sulfur fertilisation on calcium content. Schiavon et al. [50] report a decrease in calcium content in tomato leaves and no reaction in its roots under the influence of selenium fertilisation. A similar relationship was noted by Ducsay et al. [47] in wheat grains. Kopsell et al. [51] also showed that selenium supplementation did not affect calcium content in cabbage. On the other hand, Hawrylak-Nowak [52] found that the calcium content in maize shoots increased after the addition of selenium. Wu and Huang [53] also reported an increase in calcium content in fescue and clover shoots exposed to selenium. In studies by Brodowska and Kaczor [54], sulfur fertilisation resulted in an increase in calcium content in both the grain and straw of spring wheat. In another experiment by Brodowska et al. [49], the opposite reaction was observed in spring barley grain and straw. Studies on tomatoes have shown that selenium supplementation did not significantly affect calcium transport and distribution in the plant. Selenium did not alter the selectivity of Ca uptake or its allocation to leaves and roots, suggesting the stability of calcium transport pathways despite changes in the ion economy of other elements [55].

A similar relationship was reported by Ducsay et al. [47] in wheat grain, where, despite selenium fertilisation, no changes in Ca content were observed. This was explained by the low competition between selenium anions and Ca^2+^ cations and the stability of calcium transport in cereals. In the case of cabbage, it was also shown that selenium application did not affect calcium levels. Selenium modified secondary metabolism and antioxidant activity, but did not alter the concentrations of major macronutrients, including Ca [56]. Hawrylak-Nowak [52] showed an increase in Ca content in maize shoots under the influence of Se. Studies by Li et al. [57] showed that Se can improve the physiological parameters of maize, but without a confirmed increase in Ca content. Recent studies have mainly focused on the role of selenium in protecting against oxidative stress and improving enzymatic activity, but without documenting changes in calcium content.

The literature contains information on both the positive and negative effects of sulfur fertilisation on calcium content in plants. In their study, Brodowska et al. [49] showed that nutrient deficiencies (including S) can significantly affect Ca content in various cereal species. For example, increased S supply in spring barley led to a decrease in Ca content due to ion competition and changes in calcium uptake related to sulfate availability. Similar relationships were demonstrated by Barczak et al. [58], who found that sulfur fertilisation can have a bidirectional effect depending on the form of sulfur, the level of fertilisation and environmental conditions. This reflects the complex interactions between sulfur and calcium.

### 2.4. Magnesium in Wheat

The magnesium content (Table 7) in spelt wheat grain was 1.21 g kg^−1^ dry matter, and in common wheat it was 1.30 g kg^−1^ d.m. These values are considered characteristic for wheat, as studies on the mineral composition of cereal products have shown that cereals, including wheat, are an important source of magnesium in the diet and are characterised by similar Mg concentrations [58,59].

The magnesium content in wheat straw (Table 8) is also similar to the data in the literature [46]. Magnesium in plants is a component of chlorophyll and participates in photosynthesis, carbohydrate, protein and fat metabolism, and in maintaining proper ionic balance. Magnesium acts as a cofactor for numerous enzymes, stabilises ribosomes, enables ATP binding and is responsible for regulating ion transport, including Ca^2+^, K^+^ and H^+^ [26].

The application of a higher dose of selenium was associated with an 8% increase in magnesium content in spelt wheat grain. The application of both lower and higher doses of selenium was associated with an increase in magnesium content in common wheat grain by 4% and 11%, respectively. The objects in which selenium was applied at a later date were characterised by a 9% lower magnesium content in spelt wheat grain. However, the timing of selenium application was not associated with significant changes in the magnesium content of common wheat grain. Fertilisation with a lower dose of sulfur resulted in an 8% increase in magnesium content in spelt wheat grain. However, no statistically significant or clearly directed changes in magnesium content were observed in common wheat grain depending on the varying sulfur fertilisation.

The application of selenium at a lower dose resulted in a 9% reduction in magnesium content in spelt wheat straw. Selenium fertilisation, on the other hand, was associated with a significant increase in magnesium content in common wheat straw by 17% and 13%, respectively. Selenium fertilisation at a later date caused a 12% reduction in magnesium content in spelt wheat straw. The timing of selenium application did not result in clearly directed changes in magnesium content in common wheat straw. The application of a lower dose of sulfur was associated with a 15% higher magnesium content in spelt wheat straw. Sulfur fertilisation of common wheat did not cause any clear changes in the magnesium content of straw.

Ducsay et al. [47] report a significant increase in magnesium content in wheat grain under the influence of selenate (VI) fertilisation. In studies by Łukaszewicz et al. [60], selenium fertilisation resulted in a decrease in magnesium content in peas. A similar relationship was demonstrated by Hawrylak-Nowak et al. [61], who found that fertilisation with selenite (IV) resulted in a decrease in magnesium content in cucumbers, while no such reaction was observed after the application of selenate (VI). Schiavon et al. [50] did not observe any effect of selenium on the magnesium content in tomato roots and leaves. In a study by Zhang et al. [55], they confirmed that selenium had no significant effect on Mg transport and distribution in tomatoes. Selenium did not alter the selective uptake of Ca, K, Na and Mg ions or their translocation from roots to leaves. In more recent studies on plants grown in selenium-rich soils, Li et al. [57] found that magnesium content increased in the shoots of dicotyledonous plants (e.g., rapeseed and ketmia), while it decreased in the above-ground parts of grass species such as tall fescue. These results were attributed to differences in selenium tolerance, mechanisms limiting Se accumulation, and different cation management strategies in monocotyledonous and dicotyledonous plants. However, selenium did not significantly affect the magnesium content in maize, as demonstrated in studies confirming the absence of disturbances in Mg transport under the influence of selenium, even under stressful conditions [57]. Similarly, in pink catarrh [55], in shoots of reed fescue and white clover [57], no effect of selenium application on Mg content was observed.

### 2.5. Correlation Coefficients

**Figure 1:**
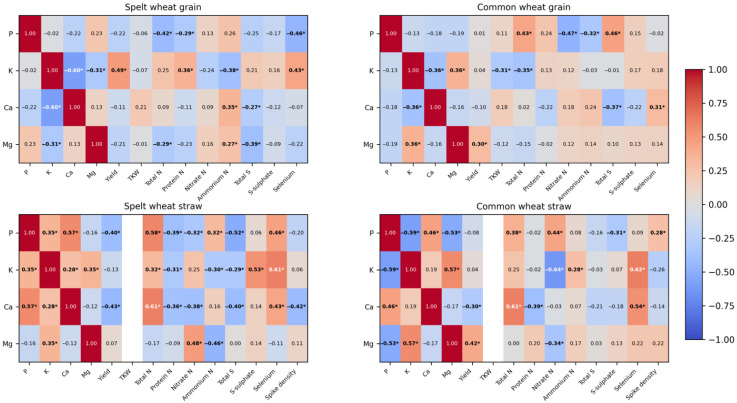
Correlation coefficients between the phosphorus, potassium, calcium and magnesium contents in plants and the chemical composition of plants and certain yield parameters of spelt and common wheat, presented in the form of a heatmap. Explanations: colours = Pearson’s correlation coefficient r (from −1 to +1); r = values in cells; asterisk (*) indicates *p* ≤ 0.05 for N = 54; TKW—thousand-kernel weight.

For the field experiment, Pearson’s linear correlation coefficients were calculated between the phosphorus, potassium, calcium and magnesium contents in plants and the chemical composition of plants and certain yield parameters of spelt wheat and common wheat (Figure 1). Detailed results concerning plant yield and its parameters, the content of total nitrogen and its fractions, and selenium and sulfur compounds can be found in previously published works [62,63,64].

A number of significant relationships between macroelements and yield components were observed in spelt wheat grain. A strong negative correlation between potassium and calcium (r = −0.60) indicates the antagonistic nature of their uptake, while the positive relationship between potassium and yield (r = 0.49) suggests a positive role for K as a factor promoting grain biomass formation. The interactions of potassium and phosphorus with selenium and nitrogen reflect the complex relationships between mineral nutrition and other element metabolism: P showed a negative correlation with Se (r = −0.46), and K showed a positive correlation (r = 0.43). Other relationships were weaker, although sometimes significant.

In common wheat grain, relationships related to phosphorus and the influence of potassium on nutrient content were dominant. Phosphorus showed strong negative relationships with the nitrate form of nitrogen (r = −0.47), while it showed positive relationships with total sulfur (r = 0.46) and total nitrogen (r = 0.43). This indicates its involvement in intensifying protein sulfur metabolism.

In spelt wheat straw, correlations between macroelements and nitrogen and sulfur dominated, indicating intense metabolic processes in the vegetative tissues of plants. Potassium showed a strong positive correlation with selenium (r = 0.61) and the sulfate form of sulfur (r = 0.53). Both calcium and phosphorus correlated strongly with total nitrogen (r = 0.61 and r = 0.58, respectively), emphasising their role in regulating growth processes and the functioning of structural proteins. Numerous negative correlations of calcium with yield (r = −0.43) and ear density (r = −0.42) may reflect the redistribution of calcium to vegetative plant tissues at the expense of generative organs. At the same time, many relationships between P, K and Mg and various forms of nitrogen (both positive and negative) indicate high dynamics of nitrogen transformations in spelt straw.

In common wheat straw, correlations were more polarised, especially with regard to potassium. Potassium showed a strong negative correlation with nitrate nitrogen (r = −0.64), which may indicate intensive utilisation of NO_3_^−^ in tissues with high metabolic activity. At the same time, the positive correlation between potassium and selenium (r = 0.62) suggests an interdependence in their accumulation. Calcium, as in spelt, was strongly associated with total nitrogen (r = 0.61) and selenium (r = 0.54); however, phosphorus showed negative associations with K (r = −0.59) and Mg (r = −0.53), indicating competition in plant uptake. Phosphorus also had numerous associations with forms of nitrogen, including a positive correlation with NO_3_^−^ (r = 0.44) and total N (r = 0.38). The relationship between Mg and straw yield (r = 0.42) and the numerous weaker correlations with yield parameters emphasise the role of magnesium in shaping wheat straw productivity.

**Figure 2:**
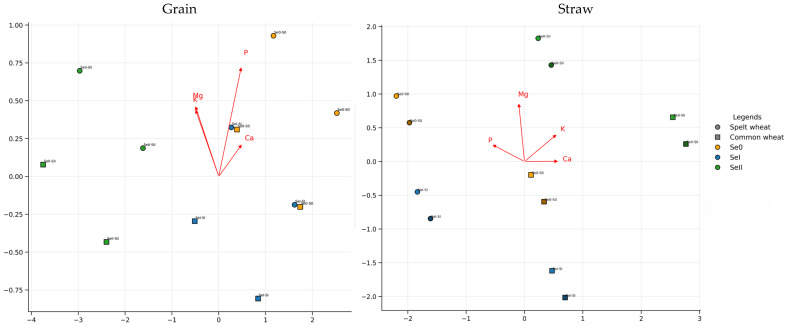
Results of the redundancy analysis (RDA) for the grain (**left panel**) and straw (**right panel**) of spelt and common wheat. The points represent combinations of selenium doses (Se0, SeI, SeII), sulfur doses (S0, SI, SII) and selenium application dates (1st, 2nd). The convex hulls correspond to the Se × application date groups. The vector arrows illustrate the direction and strength of the influence of the variables P, K, Ca and Mg on the ordination structure.

Redundancy analyses (RDA) carried out on grain and straw revealed clear and systematic variations in plant responses to selenium doses. Plants of both species (spelt and common wheat) responded differently in terms of mineral composition (Figure 2). In the case of grain, the main factors determining the ordination structure were selenium doses (Se0, SeI, and SeII), as well as their interaction with the application date. This is reflected by the clearly separated Se × application groups. The P and K variables were primarily responsible for the separation of samples along the first RDA axis, whilst Ca, particularly in common wheat, strongly influenced the dispersion along subsequent dimensions. Straw responded in a more homogeneous manner, whilst retaining the same hierarchy of factors. Selenium dose remained the main differentiating factor, although the intensity of the response was lower than in grain, and the separation of clusters was less pronounced. The P and K variables also dominated in straw, while Mg and Ca were more responsible for the subtle differentiation between factor combinations. In both analysed tissues, the interactions between the factors Se × S and Se × application date modified the position of the samples in the RDA space. However, the effect of sulfur was relatively weak and manifested itself primarily as a modulation of the response within individual Se doses. There was also a clear difference between the two species: common wheat exhibited greater variability in response and wider ordination envelopes in both grain and straw, whereas spelt wheat was characterised by a more compact distribution of data points. A comparison of the results for both plant parts indicates that whilst the grain responds to selenium fertilisation more strongly and in a more complex manner, the straw remains more stable and exhibits a milder response. This reflects the different dynamics of mineral accumulation in the generative and vegetative parts of the plant.

Aspel et al. [65] highlight the impact of sulfur on reducing the content of mineral forms of nitrogen. This results, among other things, from an improved efficiency of nitrogen uptake and utilisation and reduced losses in the form of nitrates. Similar conclusions have been drawn in a more recent meta-analysis [66], which indicates that adequate sulfur levels improve the N:S ratio, promoting more efficient nitrogen utilisation and limiting excessive accumulation of inorganic forms of N in plant tissues. This is due to the role of sulfur in plants, as it is essential for the proper functioning of enzymes responsible for nitrate reduction (V), including nitrate reductase and enzymes of the cysteine and methionine biosynthesis pathway. The availability of sulfur determines the formation of O-acetylserine and the functioning of the cysteine synthase complex, whose activity determines the efficient conversion of nitrates into organic forms [67].

In our own research, this hypothesis is confirmed by a positive correlation between the total sulfur and protein nitrogen contents in common wheat grain and spelt wheat straw. Such a positive correlation was also found in spelt wheat grain, but with a slightly lower strength.

Sulfur and selenium are chemically similar, which means that both elements are absorbed, metabolised and transported in the same way, using similar mechanisms, primarily through sulfate transporters and common stages of the sulfate/selenate reduction pathway [68]. Furthermore, sulfur deficiency leads to increased expression of enzymes involved in Se/S metabolism, such as adenylylsulfate (APS) reductase and cysteine synthase. This promotes increased uptake of selenates and their faster reduction in plant tissues [69]. According to Abdalla et al. [68] and Silva et al. [69], an increased phosphorus content in the soil can reduce the availability of selenium in the soil solution. This is due to antagonistic interactions, such as the competitive adsorption of phosphate and selenate anions, and changes in the pH and surface charge of soil sorbents. Ultimately, this limits the uptake of selenium by plants.

These studies are of crucial importance as they demonstrate that selenium fertilisation significantly alters the uptake of macronutrients and that plant responses vary markedly between species, necessitating a precise approach to biofortification. The demonstrated non-linear dose effects highlight the need to determine safe and effective Se levels, as even slight differences can lead to opposing physiological responses. Variability resulting from the timing of application indicates that further research should identify the most sensitive stages of plant development for optimal nutrient uptake. Interactions between selenium and sulfur, as well as differences between common wheat and spelt, suggest a need for future research into ion transport mechanisms and tolerance to Se- and S-induced stress. In subsequent stages of research, it will also be necessary to develop species- and variety-specific fertilisation strategies that simultaneously ensure a stable nutrient balance and high biofortification efficiency.

## 3. Materials and Methods

### 3.1. Study Design

The research was conducted based on a three-year field experiment at the Experimental Station of the University of Life Sciences in Lublin (Poland), located in Czesławice (51°18′23″ N, 22°16′02″ E). The experiment was set up in clay soil, which was formed from loess. It was characterised by elevated concentrations of phosphorus (105.5 mg P kg^−1^ d.m.) and magnesium (59.0 mg kg^−1^ d.m.), as well as moderate sulfur (10.4 mg SO_4_ kg^−1^ d.m.) and potassium (134.5 mg K kg^−1^ d.m.) contents. This soil was classified according to the IUSS Working Group WRB [70] system. The chemical characteristics of the soil substrate were presented in an earlier publication on the same research cycle [64]. The experiment used two cereal species with different functional properties: winter spelt wheat (*Triticum spelta* L., Rokosz variety) and winter common wheat (*Triticum aestivum* L., Astoria variety).

The experiment had a three-factor design. The first factor included three levels of sulfur fertilisation: S_0_—0 kg S ha^−1^, S_1_—15 kg S ha^−1^ and S_2_—30 kg S ha^−1^. The second factor included three levels of selenium supplementation: Se_0_—0 g Se ha^−1^, Se_1_—10 g Se ha^−1^ and Se_2_—20 g Se ha^−1^. The third factor was the timing of selenium application, which was carried out in two phenological intervals: during tillering (BBCH 22–24) and during the stem-elongation phase (BBCH 31–34). The variation in three levels of sulfur and selenium supply and two application dates enabled an analysis of interactions between individual environmental and agrotechnical variables.

Sowing was performed during the first ten days of October, and the crop was harvested in the second ten days of August, once the plants had reached full physiological maturity. Sulfur fertilisation was applied before sowing in the form of ammonium sulfate ((NH_4_)_2_SO_4_), while sodium selenite (Na_2_SeO_3_) served as the selenium source. An initial nitrogen dose of 20 kg N ha^−1^ (as NH_4_NO_3_) was supplied before sowing, and the remaining amount, completing a total of 80 kg N ha^−1^, was administered in two subsequent stages: 40 kg N ha^−1^ at the BBCH 22–24 growth phase (including nitrogen from ammonium sulfate) and 20 kg N ha^−1^ at BBCH 31–34. Phosphorus and potassium were both applied once before sowing, at rates of 60 kg P_2_O_5_ ha^−1^ and 80 kg K_2_O ha^−1^, respectively. Meteorological conditions and detailed information on plant-protection treatments are provided in earlier publications describing this experimental setup [63,64].

After the end of vegetation, representative samples of plant material, both grain and straw, were obtained and then subjected to laboratory analysis in accordance with the applicable chemical-analysis procedures.

### 3.2. Methods of Analysis and Statistical Evaluation

During harvesting, plant material intended for further laboratory analysis was obtained from a designated section of each experimental-plot combination (with an area of 0.25 m^2^). After mechanical separation of the grain and straw fractions, they were dried at 60 °C until a constant weight was achieved. The dried material was then ground using an electric mill to obtain a homogeneous analytical sample.

The resulting ground product (comprising both grain and straw) was digested in concentrated sulfuric acid (VI) with the addition of hydrogen peroxide acting as an oxidising catalyst. The phosphorus content in the prepared solutions was determined by a colourimetric method using vanadium–molybdenum reagents in accordance with the procedure described by Ostrowska et al. [71]. The potassium, magnesium and calcium contents were determined using atomic absorption spectrometry (AAS) according to the same methodological recommendations.

The collected numerical data were subjected to statistical analysis using three-factor analysis of variance (ANOVA) implemented in the Statistica computing environment [72]. Significant differences between the mean values of the analysed characteristics were identified using Tukey’s multiple comparison test (HSD) with a significance level of *p* ≤ 0.05 [73]. Based on the test results, the least significant difference (LSD) was determined, and homogeneous groups were identified in terms of the variability of the tested parameters (for averages over three years). In the tables presenting the interactions of experimental factors A × B × C, identical letter designations indicate the absence of statistically significant differences between the mean values, confirming their belonging to the same homogeneous group in the post hoc procedure. The convergence of letter designations clearly confirms that there are no grounds for rejecting the hypothesis of equality of means in the evaluated factor combinations. This proves their statistical equivalence with regard to the analysed feature.

## 4. Conclusions

The content of the tested macroelements was at an optimal level, and the mineral fertilisation used helped to maintain optimal plant nutrition.

Selenium fertilisation reduced the phosphorus, potassium and calcium contents in most objects of the test plants. The reactions of both wheat species to selenium application were clearly species-specific and specific to grain and straw. In common wheat grain, selenium caused an increase in K content by 4% (Se_1_) and 9% (Se_2_), as well as an increase in Mg accumulation by 4% (Se_1_) and 11% (Se_2_). In spelt wheat grain, however, a decrease in Ca accumulation by 14–18% was observed. In spelt wheat straw, selenium reduced K content by 11% (Se_1_) and Ca content by 10% (Se_1_) and 8% (Se_2_). In common wheat straw, selenium increased Mg accumulation by 17% (Se_1_) and 13% (Se_2_). Common wheat showed numerous stimulating reactions, while spelt wheat most often showed inhibitory reactions. The application of selenium generated non-linear (reversed) effects, depending on the dose, which was particularly evident for Ca and P. In common wheat straw, Se_1_ increased the P content by 11%, while Se_2_ reduced it by 5%. In common wheat grain, Se_1_ reduced Ca, while Se_2_ increased it by 10%.

The timing of selenium application did not result in a clear response from the plants. In common wheat grain, later Se application increased Ca by 11%. In spelt wheat straw, earlier application of Se increased the P content, while later application reduced Mg by 12%. The effect of the timing of selenium application depended on the element and species; earlier application had a beneficial effect on P (spelt wheat), and later application had a beneficial effect on Ca (common wheat).

Sulfur had a beneficial effect on the accumulation of phosphorus, calcium and magnesium in spelt wheat straw (positive effect), while the negative effect prevailed in common wheat straw. The content of P in spelt straw increased by 5% (S_1_) and 10% (S_2_), the content of Ca increased by 11% (S_2_), and the content of Mg increased by 15% (S_1_). In contrast, in common wheat straw, sulfur reduced K by 5% (S_1_) and Ca by 23% (S_1_) and 9% (S_2_). Sulfur has a stronger stimulating effect in spelt wheat and a more inhibitory effect in common wheat.

These findings are important because they show that selenium can substantially modify nutrient-uptake dynamics, thereby reshaping the nutritional equilibrium in wheat. The contrasting, species-dependent reactions highlight fundamental differences between common wheat and spelt in terms of ion transport regulation and their tolerance to selenium-related stress. The observed non-linear responses suggest that even minor adjustments in selenium dosage can lead to markedly different nutrient-allocation patterns, which is essential for establishing safe and effective fertilisation practices. The role of application timing is also underscored, as the developmental stage of the plant strongly influences nutrient assimilation, helping to determine the most suitable periods for selenium supply. Collectively, these outcomes underline the need for species-specific fertilisation strategies that safeguard nutrient balance while enhancing the efficiency of biofortification.

Future research should investigate why common wheat tends to exhibit stimulatory responses while spelt shows inhibitory ones, and how these contrasting behaviours stem from differences in selenium and sulfur speciation and the functioning of anion and cation transport systems.

## Figures and Tables

**Table 1:** Content of phosphorus (g kg^−1^ d.m.) in grain of spelt wheat and common wheat.

Selenium Dose (B)	Sulfur Dose (A)												Average			
	S0				SI				SII							
	I Year	II Year	III Year	$\bar{x}$	I Year	II Year	III Year	$\bar{x}$	I Year	II Year	III Year	$\bar{x}$	I Year	II Year	III Year	$\bar{x}$
Spelt wheat																
1st selenium application date (C)																
Se0	3.65	4.08	3.74	3.82 ^a^	3.75	3.59	3.26	3.53 ^a^	3.69	3.79	3.80	3.76 ^a^	3.70	3.82	3.60	3.70
SeI	3.31	3.72	3.49	3.51 ^a^	3.56	3.78	3.43	3.59 ^a^	3.72	3.74	3.60	3.69 ^a^	3.53	3.74	3.51	3.60
SeII	3.49	3.91	3.55	3.65 ^a^	3.37	3.80	3.41	3.53 ^a^	3.55	3.74	3.46	3.58 ^a^	3.47	3.82	3.47	3.59
$\bar{x}$	3.48	3.90	3.59	3.66	3.56	3.72	3.37	3.55	3.65	3.75	3.62	3.68	3.56	3.79	3.53	3.63
2nd selenium application date (C)																
Se0	3.65	4.08	3.74	3.82 ^a^	3.75	3.59	3.26	3.53 ^a^	3.69	3.79	3.80	3.76 ^a^	3.70	3.82	3.60	3.70
SeI	3.48	3.70	3.45	3.54 ^a^	3.74	3.72	3.40	3.62 ^a^	3.69	3.92	3.45	3.69 ^a^	3.63	3.78	3.43	3.62
SeII	3.89	3.81	3.05	3.58 ^a^	3.53	3.70	3.44	3.56 ^a^	3.32	3.74	3.42	3.50 ^a^	3.58	3.75	3.30	3.54
$\bar{x}$	3.67	3.86	3.41	3.65	3.67	3.67	3.37	3.57	3.57	3.82	3.56	3.65	3.64	3.78	3.45	3.62
Average																
Se0	3.65	4.08	3.74	3.82	3.75	3.59	3.26	3.53	3.69	3.79	3.80	3.76	3.70	3.82	3.60	3.70
SeI	3.40	3.71	3.47	3.53	3.65	3.75	3.42	3.61	3.71	3.83	3.53	3.69	3.58	3.76	3.47	3.61
SeII	3.69	3.86	3.30	3.62	3.45	3.75	3.43	3.55	3.44	3.74	3.44	3.54	3.53	3.79	3.39	3.57
$\bar{x}$	3.58	3.88	3.50	3.66	3.62	3.70	3.37	3.56	3.61	3.79	3.59	3.67	3.60	3.79	3.49	3.63
LSD_0.05_	A—n.s., B—0.12, C—n.s., A × B—0.29, A × C—n.s., B × C—0.04, A × B × C—n.s.															
Common wheat																
1st selenium application date (C)																
Se0	4.06	3.84	4.05	3.98 ^a^	4.08	3.51	3.90	3.83 ^a^	3.93	3.89	4.08	3.97 ^a^	4.02	3.75	4.01	3.93
SeI	4.15	3.88	4.05	4.03 ^a^	4.22	3.76	3.93	3.97 ^a^	4.20	3.78	4.16	4.05 ^a^	4.19	3.81	4.05	4.02
SeII	3.79	3.74	3.87	3.80 ^a^	3.94	3.71	3.89	3.85 ^a^	3.72	3.99	3.82	3.84 ^a^	3.82	3.81	3.86	3.83
$\bar{x}$	4.00	3.82	3.99	3.94	4.08	3.66	3.91	3.88	3.95	3.88	4.02	3.95	4.01	3.79	3.97	3.92
2nd selenium application date (C)																
Se0	4.06	3.84	4.05	3.98 ^a^	4.08	3.51	3.90	3.83 ^a^	3.93	3.89	4.08	3.97 ^a^	4.02	3.75	4.01	3.93
SeI	4.17	3.90	3.81	3.96 ^a^	4.10	3.87	4.12	4.03 ^a^	3.94	3.67	3.97	3.86 ^a^	4.07	3.81	3.97	3.95
SeII	3.93	3.95	3.75	3.87 ^a^	3.81	3.68	3.72	3.73 ^a^	3.85	3.95	3.95	3.92 ^a^	3.86	3.86	3.80	3.84
$\bar{x}$	4.05	3.89	3.87	3.94	4.00	3.68	3.91	3.86	3.91	3.84	4.00	3.92	3.98	3.81	3.93	3.91
Average																
Se0	4.06	3.84	4.05	3.98	4.08	3.51	3.90	3.83	3.93	3.89	4.08	3.97	4.02	3.75	4.01	3.93
SeI	4.16	3.89	3.93	4.00	4.16	3.82	4.03	4.00	4.07	3.73	4.07	3.96	4.13	3.81	4.01	3.99
SeII	3.86	3.85	3.81	3.84	3.88	3.70	3.81	3.79	3.79	3.97	3.89	3.88	3.84	3.84	3.83	3.84
$\bar{x}$	4.03	3.86	3.93	3.94	4.04	3.67	3.91	3.87	3.93	3.86	4.01	3.94	4.00	3.80	3.95	3.92
LSD_0.05_	A—n.s., B—0.12, C—n.s., A × B—n.s., A × C—n.s., B × C—n.s., A × B × C—n.s.															

Values marked with the same letters do not differ significantly (*p* ≤ 0.05). LSD for: A—sulfur dose, B—selenium dose, C—selenium application date; A × B, A × C, B × C, A × B × C—interactions; significant differences at *p* ≤ 0.05; n.s.—differences not significant.

**Table 2:** Content of phosphorus (g kg^−1^ d.m.) in straw of spelt wheat and common wheat.

Selenium Dose (B)	Sulfur Dose (A)												Average			
	S0				SI				SII							
	I Year	II Year	III Year	$\bar{x}$	I Year	II Year	III Year	$\bar{x}$	I Year	II Year	III Year	$\bar{x}$	I Year	II Year	III Year	$\bar{x}$
Spelt wheat																
1st selenium application date (C)																
Se0	1.52	1.34	1.42	1.42 ^a^	1.89	1.13	1.42	1.48 ^a^	2.38	1.18	1.31	1.62 ^a^	1.93	1.21	1.38	1.51
SeI	1.80	1.38	1.08	1.42 ^a^	1.33	1.18	1.64	1.38 ^a^	1.19	1.23	1.38	1.26 ^a^	1.44	1.26	1.36	1.36
SeII	2.31	0.88	1.52	1.57 ^a^	1.87	0.83	1.77	1.49 ^a^	2.17	0.76	1.68	1.54 ^a^	2.12	0.82	1.66	1.53
$\bar{x}$	1.88	1.20	1.34	1.47	1.70	1.05	1.61	1.45	1.91	1.06	1.46	1.48	1.83	1.10	1.47	1.47
2nd selenium application date (C)																
Se0	1.52	1.34	1.42	1.42 ^a^	1.89	1.13	1.42	1.48 ^a^	2.38	1.18	1.31	1.62 ^a^	1.93	1.21	1.38	1.51
SeI	1.81	0.66	1.51	1.33 ^a^	1.73	0.96	1.44	1.38 ^a^	2.13	1.12	1.56	1.60 ^a^	1.89	0.91	1.50	1.43
SeII	1.09	0.81	1.28	1.06 ^a^	1.62	1.09	1.56	1.42 ^a^	1.47	1.14	1.44	1.35 ^a^	1.40	1.01	1.43	1.28
$\bar{x}$	1.47	0.94	1.40	1.27	1.75	1.06	1.47	1.43	1.99	1.15	1.44	1.53	1.74	1.05	1.44	1.41
Average																
Se0	1.52	1.34	1.42	1.42	1.89	1.13	1.42	1.48	2.38	1.18	1.31	1.62	1.93	1.21	1.38	1.51
SeI	1.81	1.02	1.30	1.38	1.53	1.07	1.54	1.38	1.66	1.18	1.47	1.43	1.67	1.09	1.43	1.40
SeII	1.70	0.85	1.40	1.32	1.75	0.96	1.67	1.46	1.82	0.95	1.56	1.45	1.76	0.92	1.55	1.41
$\bar{x}$	1.68	1.07	1.37	1.37	1.73	1.06	1.54	1.44	1.95	1.11	1.45	1.51	1.79	1.08	1.46	1.44
LSD_0.05_	A—n.s., B—n.s., C—n.s., A × B—n.s., A × C—n.s., B × C—n.s., A × B × C—n.s.															
Common wheat																
1st selenium application date (C)																
Se0	0.98	0.94	1.56	1.16 ^a^	1.22	0.80	1.89	1.30 ^a^	1.10	0.95	1.63	1.23 ^a^	1.10	0.90	1.69	1.23
SeI	1.33	1.23	1.57	1.38 ^a^	1.43	1.01	1.98	1.47 ^a^	1.40	1.03	1.84	1.42 ^a^	1.39	1.09	1.80	1.43
SeII	0.80	0.72	1.95	1.16 ^a^	1.08	0.72	1.74	1.18 ^a^	1.00	0.76	1.54	1.10 ^a^	0.96	0.73	1.74	1.14
$\bar{x}$	1.04	0.97	1.69	1.23	1.24	0.84	1.87	1.32	1.17	0.91	1.67	1.25	1.15	0.91	1.74	1.27
2nd selenium application date (C)																
Se0	0.98	0.94	1.56	1.16 ^a^	1.22	0.80	1.89	1.30 ^a^	1.10	0.95	1.63	1.23 ^a^	1.10	0.90	1.69	1.23
SeI	1.10	0.73	2.06	1.29 ^a^	1.32	0.90	1.94	1.39 ^a^	0.98	0.95	1.74	1.22 ^a^	1.13	0.86	1.91	1.30
SeII	1.10	0.69	1.68	1.16 ^a^	1.29	0.79	1.87	1.32 ^a^	0.92	0.83	1.65	1.14 ^a^	1.10	0.77	1.73	1.20
$\bar{x}$	1.06	0.79	1.76	1.20	1.28	0.83	1.90	1.34	1.00	0.91	1.67	1.19	1.11	0.84	1.78	1.24
Average																
Se0	0.98	0.94	1.56	1.16	1.22	0.80	1.89	1.30	1.10	0.95	1.63	1.23	1.10	0.90	1.69	1.23
SeI	1.22	0.98	1.82	1.34	1.38	0.96	1.96	1.43	1.19	0.99	1.79	1.32	1.26	0.98	1.86	1.37
SeII	0.95	0.71	1.82	1.16	1.19	0.76	1.81	1.25	0.96	0.80	1.60	1.12	1.03	0.75	1.74	1.17
$\bar{x}$	1.05	0.88	1.73	1.22	1.26	0.84	1.89	1.33	1.09	0.91	1.67	1.22	1.13	0.88	1.76	1.26
LSD_0.05_	A—n.s., B—n.s., C—n.s., A × B—n.s., A × C—n.s., B × C—n.s., A × B × C—n.s.															

Explanations under Table 1.

**Table 3:** Content of potassium (g kg^−1^ d.m.) in grain of spelt wheat and common wheat.

Selenium Dose (B)	Sulfur Dose (A)												Average			
	S0				SI				SII							
	I Year	II Year	III Year	$\bar{x}$	I Year	II Year	III Year	$\bar{x}$	I Year	II Year	III Year	$\bar{x}$	I Year	II Year	III Year	$\bar{x}$
Spelt wheat																
1st selenium application date (C)																
Se0	5.36	4.87	3.11	4.45 ^a^	5.33	4.89	3.25	4.49 ^a^	5.07	4.98	3.36	4.47 ^a^	5.25	4.91	3.24	4.47
SeI	5.21	4.79	3.50	4.50 ^a^	5.35	5.11	3.41	4.62 ^a^	5.21	4.51	3.33	4.35 ^a^	5.26	4.80	3.41	4.49
SeII	5.30	4.88	3.17	4.45 ^a^	4.78	5.21	3.79	4.59 ^a^	5.30	5.16	3.24	4.57 ^a^	5.13	5.08	3.40	4.54
$\bar{x}$	5.29	4.85	3.26	4.47	5.15	5.07	3.48	4.57	5.19	4.88	3.31	4.46	5.21	4.93	3.35	4.50
2nd selenium application date (C)																
Se0	5.36	4.87	3.11	4.45 ^a^	5.33	4.89	3.25	4.49 ^a^	5.07	4.98	3.36	4.47 ^a^	5.25	4.91	3.24	4.47
SeI	5.21	4.93	3.15	4.43 ^a^	5.39	4.39	3.19	4.32 ^a^	4.94	5.23	3.35	4.51 ^a^	5.18	4.85	3.23	4.42
SeII	5.03	4.95	3.28	4.42 ^a^	4.53	4.98	3.22	4.24 ^a^	4.93	5.02	3.39	4.45 ^a^	4.83	4.98	3.30	4.37
$\bar{x}$	5.20	4.92	3.18	4.43	5.08	4.75	3.22	4.35	4.98	5.08	3.37	4.47	5.09	4.92	3.26	4.42
Average																
Se0	5.36	4.87	3.11	4.45	5.33	4.89	3.25	4.49	5.07	4.98	3.36	4.47	5.25	4.91	3.24	4.47
SeI	5.21	4.86	3.33	4.47	5.37	4.75	3.30	4.47	5.08	4.87	3.34	4.43	5.22	4.83	3.32	4.46
SeII	5.17	4.92	3.23	4.44	4.66	5.10	3.51	4.42	5.12	5.09	3.32	4.51	4.98	5.03	3.35	4.46
$\bar{x}$	5.25	4.89	3.22	4.45	5.12	4.91	3.35	4.46	5.09	4.98	3.34	4.47	5.15	4.93	3.31	4.46
LSD_0.05_	A—n.s., B—n.s., C—n.s., A × B—n.s., A × C—n.s., B × C—n.s., A × B × C—n.s.															
Common wheat																
1st selenium application date (C)																
Se0	4.92	5.05	3.21	4.39 ^a^	4.89	4.87	3.19	4.32 ^a^	5.01	4.95	3.07	4.34 ^a^	4.94	4.96	3.16	4.35
SeI	5.29	4.82	2.96	4.36 ^a^	5.09	4.71	2.09	3.96 ^a^	5.27	4.71	3.25	4.41 ^a^	5.22	4.75	2.77	4.24
SeII	6.07	5.67	2.91	4.88 ^a^	6.37	4.92	3.05	4.78 ^a^	6.33	4.87	3.22	4.81 ^a^	6.26	5.15	3.06	4.82
$\bar{x}$	5.43	5.18	3.03	4.54	5.45	4.83	2.78	4.35	5.54	4.84	3.18	4.52	5.47	4.95	2.99	4.47
2nd selenium application date (C)																
Se0	4.92	5.05	3.21	4.39 ^a^	4.89	4.87	3.19	4.32 ^a^	5.01	4.95	3.07	4.34 ^a^	4.94	4.96	3.16	4.35
SeI	6.16	5.07	3.05	4.76 ^a^	6.07	4.97	3.22	4.75 ^a^	6.25	4.95	3.20	4.80 ^a^	6.16	5.00	3.16	4.77
SeII	6.44	5.03	3.15	4.87 ^a^	5.46	4.69	3.13	4.43 ^a^	6.17	4.43	3.15	4.58 ^a^	6.02	4.72	3.14	4.63
$\bar{x}$	5.84	5.05	3.14	4.68	5.47	4.84	3.18	4.50	5.81	4.78	3.14	4.58	5.71	4.89	3.15	4.58
Average																
Se0	4.92	5.05	3.21	4.39	4.89	4.87	3.19	4.32	5.01	4.95	3.07	4.34	4.94	4.96	3.16	4.35
SeI	5.73	4.95	3.01	4.56	5.58	4.84	2.66	4.36	5.76	4.83	3.23	4.61	5.69	4.88	2.97	4.51
SeII	6.26	5.35	3.03	4.88	5.92	4.81	3.09	4.61	6.25	4.65	3.19	4.70	6.14	4.94	3.10	4.73
$\bar{x}$	5.64	5.12	3.09	4.61	5.46	4.84	2.98	4.43	5.68	4.81	3.16	4.55	5.59	4.92	3.07	4.53
LSD_0.05_	A—n.s., B—n.s., C—n.s., A × B—n.s., A × C—n.s., B × C—n.s., A × B × C—n.s.															

Explanations under Table 1.

**Table 4:** Content of potassium (g kg^−1^ d.m.) in straw of spelt wheat and common wheat.

Selenium Dose (B)	Sulfur Dose (A)												Average			
	S0				SI				SII							
	I Year	II Year	III Year	$\bar{x}$	I Year	II Year	III Year	$\bar{x}$	I Year	II Year	III Year	$\bar{x}$	I Year	II Year	III Year	$\bar{x}$
Spelt wheat																
1st selenium application date (C)																
Se0	9.32	10.64	6.22	8.73 ^a^	8.04	10.24	6.09	8.12 ^a^	8.35	12.46	4.90	8.57 ^a^	8.57	11.11	5.74	8.47
SeI	7.73	10.59	5.75	8.02 ^a^	6.86	8.45	5.62	6.98 ^a^	8.72	9.36	6.53	8.20 ^a^	7.77	9.47	5.97	7.73
SeII	10.53	8.88	5.52	8.31 ^a^	11.39	6.91	5.00	7.77 ^a^	13.43	9.25	2.84	8.51 ^a^	11.78	8.35	4.45	8.19
$\bar{x}$	9.19	10.04	5.83	8.35	8.76	8.53	5.57	7.62	10.17	10.36	4.76	8.43	9.37	9.64	5.39	8.13
2nd selenium application date (C)																
Se0	9.32	10.64	6.22	8.73 ^a^	8.04	10.24	6.09	8.12 ^a^	8.35	12.46	4.90	8.57 ^a^	8.57	11.11	5.74	8.47
SeI	9.31	7.39	4.97	7.22 ^a^	10.41	8.57	4.04	7.67 ^a^	8.19	8.00	5.03	7.07 ^a^	9.30	7.99	4.68	7.32
SeII	10.23	10.55	4.26	8.35 ^a^	9.77	8.70	5.05	7.84 ^a^	15.14	7.74	4.04	8.97 ^a^	11.71	9.00	4.45	8.39
$\bar{x}$	9.62	9.53	5.15	8.10	9.41	9.17	5.06	7.88	10.56	9.40	4.66	8.21	9.86	9.37	4.96	8.06
Average																
Se0	9.32	10.64	6.22	8.73	8.04	10.24	6.09	8.12	8.35	12.46	4.90	8.57	8.57	11.11	5.74	8.47
SeI	8.52	8.99	5.36	7.62	8.64	8.51	4.83	7.33	8.46	8.68	5.78	7.64	8.54	8.73	5.33	7.53
SeII	10.38	9.72	4.89	8.33	10.58	7.81	5.03	7.81	14.29	8.50	3.44	8.74	11.75	8.68	4.45	8.29
$\bar{x}$	9.41	9.79	5.49	8.23	9.09	8.85	5.32	7.75	10.37	9.88	4.71	8.32	9.62	9.51	5.18	8.10
LSD_0.05_	A—n.s., B—n.s., C—n.s., A × B—n.s., A × C—n.s., B × C—n.s., A × B × C—n.s.															
Common wheat																
1st selenium application date (C)																
Se0	9.53	12.02	5.57	9.04 ^a^	8.56	14.31	5.51	9.46 ^a^	7.91	18.86	6.35	11.04 ^a^	8.67	15.06	5.81	9.85
SeI	9.36	12.63	5.90	9.30 ^a^	9.16	10.51	5.68	8.45 ^a^	10.66	10.94	5.50	9.03 ^a^	9.73	11.36	5.69	8.93
SeII	14.07	11.01	7.01	10.70 ^a^	10.98	8.85	3.46	7.76 ^a^	8.19	11.36	6.07	8.54 ^a^	11.08	10.41	5.51	9.00
$\bar{x}$	10.99	11.89	6.16	9.68	9.57	11.22	4.88	8.56	8.92	13.72	5.97	9.54	9.82	12.28	5.67	9.26
2nd selenium application date (C)																
Se0	9.53	12.02	5.57	9.04 ^a^	8.56	14.31	5.51	9.46 ^a^	7.91	18.86	6.35	11.04 ^a^	8.67	15.06	5.81	9.85
SeI	8.63	9.67	4.53	7.61 ^a^	10.67	8.61	7.38	8.89 ^a^	14.12	9.99	6.35	10.15 ^a^	11.14	9.42	6.09	8.88
SeII	12.85	9.78	7.31	9.98 ^a^	10.69	11.26	4.76	8.90 ^a^	9.53	10.10	5.91	8.51 ^a^	11.02	10.38	5.99	9.13
$\bar{x}$	10.34	10.49	5.80	8.88	9.97	11.39	5.88	9.08	10.52	12.98	6.20	9.90	10.28	11.62	5.96	9.29
Average																
Se0	9.53	12.02	5.57	9.04	8.56	14.31	5.51	9.46	7.91	18.86	6.35	11.04	8.67	15.06	5.81	9.85
SeI	9.00	11.15	5.22	8.46	9.92	9.56	6.53	8.67	12.39	10.47	5.93	9.59	10.44	10.39	5.89	8.91
SeII	13.46	10.40	7.16	10.34	10.84	10.06	4.11	8.33	8.86	10.73	5.99	8.53	11.05	10.40	5.75	9.07
$\bar{x}$	10.67	11.19	5.98	9.28	9.77	11.31	5.38	8.82	9.72	13.35	6.09	9.72	10.05	11.95	5.82	9.28
LSD_0.05_	A—n.s., B—n.s., C—n.s., A × B—n.s., A × C—n.s., B × C—n.s., A × B × C—n.s.															

Explanations under Table 1.

**Table 5:** Content of calcium (g kg^−1^ d.m.) in grain of spelt wheat and common wheat.

Selenium Dose (B)	Sulfur Dose (A)												Average			
	S0				SI				SII							
	I Year	II Year	III Year	$\bar{x}$	I Year	II Year	III Year	$\bar{x}$	I Year	II Year	III Year	$\bar{x}$	I Year	II Year	III Year	$\bar{x}$
Spelt wheat																
1st selenium application date (C)																
Se0	0.83	1.49	2.34	1.55 ^a^	0.94	1.46	3.39	1.93 ^a^	1.14	1.56	1.93	1.54 ^a^	0.97	1.50	2.55	1.68
SeI	0.95	1.50	2.09	1.51 ^a^	0.87	1.58	2.05	1.50 ^a^	0.91	1.44	2.05	1.47 ^a^	0.91	1.51	2.06	1.49
SeII	0.79	1.38	1.95	1.37 ^a^	0.78	1.55	1.44	1.26 ^a^	0.71	1.54	1.43	1.23 ^a^	0.76	1.49	1.61	1.29
$\bar{x}$	0.86	1.46	2.13	1.48	0.86	1.53	2.29	1.56	0.92	1.51	1.80	1.41	0.88	1.50	2.07	1.48
2nd selenium application date (C)																
Se0	0.83	1.49	2.34	1.55 ^a^	0.94	1.46	3.39	1.93 ^a^	1.14	1.56	1.93	1.54 ^a^	0.97	1.50	2.55	1.68
SeI	0.81	1.38	1.99	1.39 ^a^	0.83	1.65	1.65	1.38 ^a^	0.84	1.44	1.89	1.39 ^a^	0.83	1.49	1.84	1.39
SeII	0.84	1.62	1.89	1.45 ^a^	0.75	1.51	2.27	1.51 ^a^	0.79	1.54	1.75	1.36 ^a^	0.79	1.56	1.97	1.44
$\bar{x}$	0.83	1.50	2.07	1.47	0.84	1.54	2.44	1.61	0.92	1.51	1.86	1.43	0.86	1.52	2.12	1.50
Average																
Se0	0.83	1.49	2.34	1.55	0.94	1.46	3.39	1.93	1.14	1.56	1.93	1.54	0.97	1.50	2.55	1.68
SeI	0.88	1.44	2.04	1.45	0.85	1.62	1.85	1.44	0.88	1.44	1.97	1.43	0.87	1.50	1.95	1.44
SeII	0.82	1.50	1.92	1.41	0.77	1.53	1.86	1.39	0.75	1.54	1.59	1.30	0.78	1.53	1.79	1.37
$\bar{x}$	0.85	1.48	2.10	1.48	0.85	1.54	2.37	1.59	0.92	1.51	1.83	1.42	0.87	1.51	2.10	1.49
LSD_0.05_	A—n.s., B—0.30, C—n.s., A × B—n.s., A × C—n.s., B × C—0.10, A × B × C—n.s.															
Common wheat																
1st selenium application date (C)																
Se0	1.33	2.03	1.86	1.74 ^a^	1.16	1.97	1.89	1.67 ^a^	1.14	1.58	1.83	1.52 ^a^	1.21	1.86	1.86	1.64
SeI	1.17	1.79	1.85	1.60 ^a^	1.13	1.89	1.61	1.54 ^a^	1.08	1.76	1.98	1.61 ^a^	1.13	1.81	1.81	1.58
SeII	1.17	2.02	2.37	1.85 ^a^	1.01	1.69	1.59	1.43 ^a^	1.01	1.74	1.65	1.47 ^a^	1.06	1.82	1.87	1.58
$\bar{x}$	1.22	1.95	2.03	1.73	1.10	1.85	1.70	1.55	1.08	1.69	1.82	1.53	1.13	1.83	1.85	1.60
2nd selenium application date (C)																
Se0	1.33	2.03	1.86	1.74 ^a^	1.16	1.97	1.89	1.67 ^a^	1.14	1.58	1.83	1.52 ^a^	1.21	1.86	1.86	1.64
SeI	1.04	1.84	2.03	1.64 ^a^	1.18	1.82	2.00	1.67 ^a^	1.20	1.73	1.99	1.64 ^a^	1.14	1.80	2.01	1.65
SeII	1.26	1.77	2.05	1.69 ^a^	1.16	1.71	2.36	1.74 ^a^	0.98	1.55	5.55	2.69 ^a^	1.13	1.68	3.32	2.04
$\bar{x}$	1.21	1.88	1.98	1.69	1.17	1.83	2.08	1.69	1.11	1.62	3.12	1.95	1.16	1.78	2.40	1.78
Average																
Se0	1.33	2.03	1.86	1.74	1.16	1.97	1.89	1.67	1.14	1.58	1.83	1.52	1.21	1.86	1.86	1.64
SeI	1.11	1.82	1.94	1.62	1.16	1.86	1.81	1.61	1.14	1.75	1.99	1.63	1.14	1.81	1.91	1.62
SeII	1.22	1.90	2.21	1.77	1.09	1.70	1.98	1.59	1.00	1.65	3.60	2.08	1.10	1.75	2.60	1.81
$\bar{x}$	1.22	1.92	2.01	1.71	1.14	1.84	1.89	1.62	1.10	1.66	2.47	1.74	1.15	1.81	2.13	1.69
LSD_0.05_	A—n.s., B—n.s., C—n.s., A × B—n.s., A × C—n.s., B × C—n.s., A × B × C—n.s.															

Explanations under Table 1.

**Table 6:** Content of calcium (g kg^−1^ d.m.) in straw of spelt wheat and common wheat.

Selenium Dose (B)	Sulfur Dose (A)												Average			
	S0				SI				SII							
	I Year	II Year	III Year	$\bar{x}$	I Year	II Year	III Year	$\bar{x}$	I Year	II Year	III Year	$\bar{x}$	I Year	II Year	III Year	$\bar{x}$
Spelt wheat																
1st selenium application date (C)																
Se0	9.55	3.03	9.79	7.46 ^a^	10.23	4.30	3.16	5.90 ^a^	11.45	4.42	6.85	7.57 ^a^	10.41	3.92	6.60	6.98
SeI	11.44	3.10	9.13	7.89 ^a^	9.57	2.80	8.42	6.93 ^a^	9.05	3.49	6.68	6.41 ^a^	10.02	3.13	8.08	7.08
SeII	8.26	2.68	7.25	6.06 ^a^	10.33	3.11	4.19	5.88 ^a^	6.09	6.89	8.60	7.19 ^a^	8.23	4.23	6.68	6.38
$\bar{x}$	9.75	2.94	8.72	7.14	10.04	3.40	5.26	6.23	8.86	4.93	7.38	7.06	9.55	3.76	7.12	6.81
2nd selenium application date (C)																
Se0	9.55	3.03	9.79	7.46 ^a^	10.23	4.30	3.16	5.90 ^a^	11.45	4.42	6.85	7.57 ^a^	10.41	3.92	6.60	6.98
SeI	5.15	3.54	4.47	4.39 ^a^	10.30	3.56	7.15	7.00 ^a^	8.07	3.85	3.61	5.18 ^a^	7.84	3.65	5.08	5.52
SeII	6.77	2.41	5.43	4.87 ^a^	7.90	4.62	6.33	6.28 ^a^	6.70	6.36	12.04	8.37 ^a^	7.12	4.46	7.93	6.51
$\bar{x}$	7.16	2.99	6.56	5.57	9.48	4.16	5.55	6.39	8.74	4.88	7.50	7.04	8.46	4.01	6.54	6.33
Average																
Se0	9.55	3.03	9.79	7.46	10.23	4.30	3.16	5.90	11.45	4.42	6.85	7.57	10.41	3.92	6.60	6.98
SeI	8.30	3.32	6.80	6.14	9.94	3.18	7.79	6.97	8.56	3.67	5.15	5.80	8.93	3.39	6.58	6.30
SeII	7.52	2.55	6.34	5.47	9.12	3.87	5.26	6.08	6.40	6.63	10.32	7.78	7.68	4.35	7.31	6.45
$\bar{x}$	8.46	2.97	7.64	6.36	9.76	3.78	5.41	6.31	8.80	4.91	7.44	7.05	9.01	3.89	6.83	6.57
LSD_0.05_	A—n.s., B—n.s., C—n.s., A × B—0.06, A × C—n.s., B × C—n.s., A × B × C—n.s.															
Common wheat																
1st selenium application date (C)																
Se0	6.14	5.28	9.44	6.95 ^a^	7.39	2.53	6.58	5.50 ^a^	7.92	2.92	11.60	7.48 ^a^	7.15	3.58	9.21	6.64
SeI	9.57	7.06	6.45	7.69 ^a^	6.89	2.66	6.70	5.42 ^a^	8.17	5.19	10.43	7.93 ^a^	8.21	4.97	7.86	7.01
SeII	11.11	3.34	6.11	6.85 ^a^	8.88	2.41	7.92	6.40 ^a^	7.42	2.50	6.45	5.46 ^a^	9.14	2.75	6.83	6.24
$\bar{x}$	8.94	5.23	7.33	7.17	7.72	2.53	7.07	5.77	7.84	3.54	9.49	6.96	8.17	3.77	7.96	6.63
2nd selenium application date (C)																
Se0	6.14	5.28	9.44	6.95 ^a^	7.39	2.53	6.58	5.50 ^a^	7.92	2.92	11.60	7.48 ^a^	7.15	3.58	9.21	6.64
SeI	9.33	5.43	11.36	8.71 ^a^	7.82	2.43	7.52	5.92 ^a^	12.74	3.77	7.92	8.14 ^a^	9.96	3.88	8.93	7.59
SeII	12.93	2.40	7.95	7.76 ^a^	9.48	2.62	5.47	5.86 ^a^	6.94	2.81	3.38	4.38 ^a^	9.78	2.61	5.60	6.00
$\bar{x}$	9.47	4.37	9.58	7.81	8.23	2.53	6.52	5.76	9.20	3.17	7.63	6.67	8.97	3.35	7.91	6.74
Average																
Se0	6.14	5.28	9.44	6.95	7.39	2.53	6.58	5.50	7.92	2.92	11.60	7.48	7.15	3.58	9.21	6.64
SeI	9.45	6.25	8.91	8.20	7.36	2.55	7.11	5.67	10.46	4.48	9.18	8.04	9.09	4.43	8.40	7.30
SeII	12.02	2.87	7.03	7.31	9.18	2.52	6.70	6.13	7.18	2.66	4.92	4.92	9.46	2.68	6.22	6.12
$\bar{x}$	9.21	4.80	8.46	7.49	7.98	2.53	6.80	5.77	8.52	3.36	8.56	6.82	8.57	3.56	7.94	6.69
LSD_0.05_	A—n.s., B—n.s., C—n.s., A × B—n.s., A × C—n.s., B × C—n.s., A × B × C—n.s.															

Explanations under Table 1.

**Table 7:** Content of magnesium (g kg^−1^ d.m.) in grain of spelt wheat and common wheat.

Selenium Dose (B)	Sulfur Dose (A)												Average			
	S0				SI				SII							
	I Year	II Year	III Year	$\bar{x}$	I Year	II Year	III Year	$\bar{x}$	I Year	II Year	III Year	$\bar{x}$	I Year	II Year	III Year	$\bar{x}$
Spelt wheat																
1st selenium application date (C)																
Se0	1.07	1.27	1.34	1.23 ^a^	1.06	1.28	0.99	1.11 ^a^	1.02	1.34	1.22	1.19 ^a^	1.05	1.30	1.18	1.18
SeI	1.04	1.27	1.24	1.18 ^a^	1.10	1.32	1.56	1.33 ^a^	1.06	1.32	1.24	1.21 ^a^	1.07	1.30	1.35	1.24
SeII	1.04	1.30	1.09	1.14 ^a^	1.01	2.20	2.44	1.88 ^a^	1.06	1.27	1.18	1.17 ^a^	1.04	1.59	1.57	1.40
$\bar{x}$	1.05	1.28	1.22	1.18	1.06	1.60	1.66	1.44	1.05	1.31	1.21	1.19	1.05	1.40	1.37	1.27
2nd selenium application date (C)																
Se0	1.07	1.27	1.34	1.23 ^a^	1.06	1.28	0.99	1.11 ^a^	1.02	1.34	1.22	1.19 ^a^	1.05	1.30	1.18	1.18
SeI	1.04	1.27	1.00	1.10 ^a^	1.09	1.19	1.13	1.14 ^a^	0.99	1.32	1.00	1.10 ^a^	1.04	1.26	1.04	1.11
SeII	1.05	1.39	1.07	1.17 ^a^	0.94	1.25	1.06	1.08 ^a^	1.05	1.30	1.26	1.20 ^a^	1.01	1.31	1.13	1.15
$\bar{x}$	1.05	1.31	1.14	1.17	1.03	1.24	1.06	1.11	1.02	1.32	1.16	1.17	1.03	1.29	1.12	1.15
Average																
Se0	1.07	1.27	1.34	1.23	1.06	1.28	0.99	1.11	1.02	1.34	1.22	1.19	1.05	1.30	1.18	1.18
SeI	1.04	1.27	1.12	1.14	1.10	1.26	1.35	1.24	1.03	1.32	1.12	1.16	1.06	1.28	1.20	1.18
SeII	1.05	1.35	1.08	1.16	0.98	1.73	1.75	1.48	1.06	1.29	1.22	1.19	1.03	1.45	1.35	1.28
$\bar{x}$	1.05	1.30	1.18	1.18	1.05	1.42	1.36	1.28	1.04	1.32	1.19	1.18	1.04	1.35	1.25	1.21
LSD_0.05_	A—n.s., B—n.s., C—n.s., A × B—n.s., A × C—n.s., B × C—n.s., A × B × C—n.s.															
Common wheat																
1st selenium application date (C)																
Se0	1.16	1.27	1.23	1.22 ^a^	1.17	1.28	1.31	1.25 ^a^	1.20	1.34	1.20	1.25 ^a^	1.18	1.30	1.25	1.24
SeI	1.32	1.27	1.18	1.26 ^a^	1.22	1.32	0.79	1.11 ^a^	1.24	1.32	1.48	1.35 ^a^	1.26	1.30	1.15	1.24
SeII	1.49	1.30	1.17	1.32 ^a^	1.36	2.20	1.46	1.67 ^a^	1.41	1.27	1.20	1.29 ^a^	1.42	1.59	1.28	1.43
$\bar{x}$	1.32	1.28	1.19	1.27	1.25	1.60	1.19	1.35	1.28	1.31	1.29	1.30	1.29	1.40	1.22	1.30
2nd selenium application date (C)																
Se0	1.16	1.27	1.23	1.22 ^a^	1.17	1.28	1.31	1.25 ^a^	1.20	1.34	1.20	1.25 ^a^	1.18	1.30	1.25	1.24
SeI	1.46	1.27	1.28	1.34 ^a^	1.45	1.19	1.14	1.26 ^a^	1.54	1.32	1.31	1.39 ^a^	1.48	1.26	1.24	1.33
SeII	1.59	1.39	1.21	1.40 ^a^	1.36	1.25	1.26	1.29 ^a^	1.44	1.30	1.20	1.31 ^a^	1.46	1.31	1.22	1.33
$\bar{x}$	1.40	1.31	1.24	1.32	1.33	1.24	1.24	1.27	1.39	1.32	1.24	1.32	1.37	1.29	1.24	1.30
Average																
Se0	1.16	1.27	1.23	1.22	1.17	1.28	1.31	1.25	1.20	1.34	1.20	1.25	1.18	1.30	1.25	1.24
SeI	1.39	1.27	1.23	1.30	1.34	1.26	0.97	1.19	1.39	1.32	1.40	1.37	1.37	1.28	1.20	1.29
SeII	1.54	1.35	1.19	1.36	1.36	1.73	1.36	1.48	1.43	1.29	1.20	1.30	1.44	1.45	1.25	1.38
$\bar{x}$	1.36	1.30	1.22	1.30	1.29	1.42	1.22	1.31	1.34	1.32	1.27	1.31	1.33	1.35	1.23	1.30
LSD_0.05_	A—n.s., B—0.09, C—n.s., A × B—n.s., A × C—n.s., B × C—n.s., A × B × C—n.s.															

Explanations under Table 1.

**Table 8:** Content of magnesium (g kg^−1^ d.m.) in straw of spelt wheat and common wheat.

Selenium Dose (B)	Sulfur Dose (A)												Average			
	S0				SI				SII							
	I Year	II Year	III Year	$\bar{x}$	I Year	II Year	III Year	$\bar{x}$	I Year	II Year	III Year	$\bar{x}$	I Year	II Year	III Year	$\bar{x}$
Spelt wheat																
1st selenium application date (C)																
Se0	0.65	0.68	0.39	0.57 ^a^	0.72	0.64	0.44	0.60 ^a^	0.67	0.64	0.28	0.53 ^a^	0.68	0.65	0.37	0.57
SeI	0.61	0.62	0.31	0.51 ^a^	0.67	0.63	0.33	0.54 ^a^	0.76	0.55	0.50	0.60 a	0.68	0.60	0.38	0.55
SeII	0.64	0.52	0.48	0.55 ^a^	0.55	2.07	0.28	0.97 ^a^	0.54	0.64	0.23	0.47 ^a^	0.58	1.08	0.33	0.66
$\bar{x}$	0.63	0.61	0.39	0.54	0.65	1.11	0.35	0.70	0.66	0.61	0.34	0.53	0.65	0.78	0.36	0.59
2nd selenium application date (C)																
Se0	0.65	0.68	0.39	0.57 ^a^	0.72	0.64	0.44	0.60 ^a^	0.67	0.64	0.28	0.53 ^a^	0.68	0.65	0.37	0.57
SeI	0.49	0.61	0.36	0.49 ^a^	0.61	0.53	0.25	0.46 ^a^	0.58	0.59	0.36	0.51 ^a^	0.56	0.58	0.32	0.49
SeII	0.61	0.61	0.32	0.51 ^a^	0.60	0.48	0.35	0.48 ^a^	0.59	0.56	0.32	0.49 ^a^	0.60	0.55	0.33	0.49
$\bar{x}$	0.58	0.63	0.36	0.52	0.64	0.55	0.35	0.51	0.61	0.60	0.32	0.51	0.61	0.59	0.34	0.52
Average																
Se0	0.65	0.68	0.39	0.57	0.72	0.64	0.44	0.60	0.67	0.64	0.28	0.53	0.68	0.65	0.37	0.57
SeI	0.55	0.62	0.34	0.50	0.64	0.58	0.29	0.50	0.67	0.57	0.43	0.56	0.62	0.59	0.35	0.52
SeII	0.63	0.57	0.40	0.53	0.58	1.28	0.32	0.73	0.57	0.60	0.28	0.48	0.59	0.82	0.33	0.58
$\bar{x}$	0.61	0.62	0.38	0.53	0.65	0.83	0.35	0.61	0.64	0.61	0.33	0.52	0.63	0.69	0.35	0.56
LSD_0.05_	A—n.s., B—n.s., C—n.s., A × B—n.s., A × C—n.s., B × C—n.s., A × B × C—n.s.															
Common wheat																
1st selenium application date (C)																
Se0	0.52	0.44	0.34	0.43 ^a^	0.51	0.56	0.37	0.48 ^a^	0.54	0.52	0.39	0.48 ^a^	0.52	0.51	0.37	0.47
SeI	0.54	0.63	0.44	0.54 ^a^	0.59	0.62	0.42	0.54 ^a^	0.50	0.69	0.50	0.56 ^a^	0.54	0.65	0.45	0.55
SeII	0.55	0.67	0.54	0.59 ^a^	0.51	0.48	0.39	0.46 ^a^	0.54	0.52	0.47	0.51 ^a^	0.53	0.56	0.47	0.52
$\bar{x}$	0.54	0.58	0.44	0.52	0.54	0.55	0.39	0.49	0.53	0.58	0.45	0.52	0.53	0.57	0.43	0.51
2nd selenium application date (C)																
Se0	0.52	0.44	0.34	0.43 ^a^	0.51	0.56	0.37	0.48 ^a^	0.54	0.52	0.39	0.48 ^a^	0.52	0.51	0.37	0.47
SeI	0.54	0.62	0.54	0.57 ^a^	0.54	0.46	0.50	0.50 ^a^	0.61	0.58	0.56	0.58 ^a^	0.56	0.55	0.53	0.55
SeII	0.64	0.53	0.38	0.52 ^a^	0.56	0.60	0.53	0.56 ^a^	0.58	0.61	0.39	0.53 ^a^	0.59	0.58	0.43	0.54
$\bar{x}$	0.57	0.53	0.42	0.51	0.54	0.54	0.47	0.51	0.58	0.57	0.45	0.53	0.56	0.55	0.44	0.52
Average																
Se0	0.52	0.44	0.34	0.43	0.51	0.56	0.37	0.48	0.54	0.52	0.39	0.48	0.52	0.51	0.37	0.47
SeI	0.54	0.63	0.49	0.56	0.57	0.54	0.46	0.52	0.56	0.64	0.53	0.57	0.55	0.60	0.49	0.55
SeII	0.60	0.60	0.46	0.56	0.54	0.54	0.46	0.51	0.56	0.57	0.43	0.52	0.56	0.57	0.45	0.53
$\bar{x}$	0.56	0.56	0.43	0.52	0.54	0.55	0.43	0.50	0.56	0.58	0.45	0.53	0.55	0.56	0.44	0.52
LSD_0.05_	A—n.s., B—0.05, C—n.s., A × B—n.s., A × C—n.s., B × C—n.s., A × B × C—n.s.															

Explanations under Table 1.

## Data Availability

Data are contained within the article.

## References

[1] Brodowska M.S., Muszyński P., Haliniarz M., Brodowski R., Kowalczyk-Juśko A., Sekutowski T., Kurzyna-Szklarek M. (2018). Agronomic Aspects of Switchgrass Cultivation and Use for Energy Purposes. Appl. Ecol. Environ. Res..

[2] Bogusz P., Rusek P., Brodowska M.S. (2021). Suspension Fertilizers: How to Reconcile Sustainable Fertilization and Environmental Protection. Agriculture.

[3] Shiwakoti S., Zheljazkov V.D., Gollany H.T., Kleber M., Xing B., Astatkie T. (2020). Macronutrient in Soils and Wheat from Long-Term Agroexperiments Reflects Variations in Residue and Fertilizer Inputs. Sci. Rep..

[4] Brodowska M.S., Wyszkowski M., Kordala N. (2022). Use of Organic Materials to Limit the Potential Negative Effect of Nitrogen on Maize in Different Soils. Materials.

[5] Arlauskienė A., Petraitytė D., Palubinskas T., Jimenez M., Cesevičienė J. (2025). Nutritional Status and Nitrogen Uptake Dynamics of Waxy-Type Winter Wheat Under Liquid Organic Fertilization. Plants.

[6] Kisko M., Shukla V., Kaur M., Bouain N., Chaiwong N., Lacombe B., Pandey A.K., Rouached H. (2018). Phosphorus Transport in Arabidopsis and Wheat: Emerging Strategies to Improve P Pool in Seeds. Agriculture.

[7] Ecik B. (2026). What Is Phosphorus Fertilizer? Usage Timing, Benefits, and Types. Esular Smart Agriculture Technologies. https://esular.com/what-is-phosphorus-fertilizer-usage-timing-benefits-types.

[8] Mühlbachová G., Kusá H., Růžek P., Káš M., Vavera R. (2025). Soil Nutrient Contents in a Long-Term Field Experiment Following the Suspension of Phosphorus and Potassium Fertilisation. Plant Soil Environ..

[9] Parra-Almuna L., Pontigo S., Ruiz A., González F., Ferrol N., de la Luz Mora M., Cartes P. (2024). Dissecting the Roles of Phosphorus Use Efficiency, Organic Acid Anions, and Aluminum-Responsive Genes under Aluminum Toxicity and Phosphorus Deficiency in Ryegrass Plants. Plants.

[10] Hou P., Li B., Cao E., Liu Z., Li Y., Sun Z., Xiao Y., Ma C. (2025). Optimizing Nitrogen and Phosphorus Fertilizer Application for Wheat Yield on Alkali Soils: Mechanisms and Effects. Agronomy.

[11] Wang W., Sun M., Lin W., Ren A., Xue J., Yu S., Zhang R., Gao Z. (2021). Effects of Phosphorus Fertilizer on Root Characteristics, Uptake and Utilization of Phosphorus and Yield of Dryland Wheat with Contrasting Rainfall Patterns. Chin. J. Appl. Ecol..

[12] Raza M.A.S., Saleem M.F., Shah G.M., Jamil M., Khan I.H. (2013). Potassium Applied under Drought Improves Physiological and Nutrient Uptake Performances of Wheat (*Triticum aestivum* L.). J. Soil Sci. Plant Nutr..

[13] Biology Insights Editorial Team (2026). Where Do Plants Get Potassium From?. Biol. Insights.

[14] Sulaman S., Nadeem M., Shabaan M., Orman S., Anwar-ul-Haq M., Zulfiqar U. (2025). Exogenous Application of Nitrogen (N) and Potassium (K) Improves Drought Tolerance in Plants: A Review. J. Soil Sci. Plant Nutr..

[15] Noor F., Nawaz H., Khan A., Shani M.Y., Azmat M., Abbas S.M., Arshad I., Aziz R., Saleem M., De Mastro F. (2025). Effect of Foliar Application of Potassium on Wheat Tolerance to Salt Stress. PLoS ONE.

[16] Kibarov O., Trokhymenko G., Nedoroda V. (2025). The Effect of Biostimulants and Glyphosate on the Vertical Migration of Elements in Agricultural Soils. Ukrainian Black Sea Region Agr. Sci..

[17] Sedri M.H., Roohi E., Niazian M., Niedbała G. (2022). Interactive Effects of Nitrogen and Potassium Fertilizers on Quantitative–Qualitative Traits and Drought Tolerance Indices of Rainfed Wheat Cultivar. Agronomy.

[18] Zhang M., Du G., Zhang H., Zheng R., Zhao L., Huang M., Wang X., Liu K., Yan D. (2026). Metabolomics and Biochemical Analysis Reveal the Regulatory Mechanism of Exogenous Sorbitol Chelated Potassium on Wheat under Drought Stress. Front. Plant Sci..

[19] Dora Agri-Tech Research Group (2020). Application and Value of Fulvic Acid in Agriculture. Dora Agri-Tech. https://doraagri.com/application-and-value-of-fulvic-acid-in-agriculture/.

[20] Thor K. (2019). Calcium—Nutrient and Messenger. Front. Plant Sci..

[21] Wdowiak A., Podgórska A., Szal B. (2024). Calcium in Plants: An Important Element of Cell Physiology and Structure, Signaling, and Stress Responses. Acta Physiol. Plant..

[22] Wang T., Chen X., Ju C., Wang C. (2023). Calcium Signaling in Plant Mineral Nutrition: From Uptake to Transport. Plant Communic..

[23] Hussain Z. Soil pH: Why It Matters More Than Fertilizer for Crop Productivity. Agri Harbor Hub 2026. https://agriharborhub.blogspot.com/2026/01/soil-ph-why-it-matters-more-than.html.

[24] Kabir M.Y., Díaz Pérez J.C. (2025). Calcium Route in the Plant and Blossom-End Rot Incidence. Horticulturae.

[25] Palta J.P. (2014). Role of Calcium in Plant Responses to Stresses. Hort. Sci..

[26] Hosseini Pouya H.S., Abdullah, Heidari P. (2026). Magnesium Transport Systems in Plants: Function, Physiological Roles, and Impact on Related Molecular Pathways: A Review. Plant Mol. Biol. Rep..

[27] Jia X., Wang Y., Wang T., Zhu B., Weng Q., Liao Y., Gu J., Luo Y., Zhang Q., Ye J. (2026). Response of Characteristic Hormones in Tea Roots and Leaves under Magnesium Regulation and Their Balancing Regulation on Growth and Quality. Front. Plant Sci..

[28] Saquee F.S., Diakite S., Kavhiza N.J., Pakina E., Zargar M. (2023). The Efficacy of Micronutrient Fertilizers on the Yield Formulation and Quality of Wheat Grains. Agronomy.

[29] Sherefu A., Zewide I. (2021). Review Paper on Effect of Micronutrients for Crop Production. J. Nutr. Food Process..

[30] Gupta M., Gupta S. (2017). An Overview of Selenium Uptake, Metabolism, and Toxicity in Plants. Front. Plant Sci. Sec. Plant Membr. Traffic Transp..

[31] Pilon-Smits E.A.H., Quinn C.F., Hell R., Mendel R.-R. (2010). Selenium Metabolism in Plants. Cell Biology of Metals and Nutrients.

[32] Trippe R.C., Pilon-Smits E.A.H. (2021). Selenium Transport and Metabolism in Plants: Phytoremediation and Biofortification Implications. J. Hazard. Mater..

[33] White P.J., Bowen H.C., Parmaguru P., Fritz M., Spracklen W.P., Spiby R.E., Meacham M.C., Mead A., Harriman M., Trueman L.J. (2004). Interactions Between Selenium and Sulphur Nutrition in Arabidopsis thaliana. J. Exp. Bot..

[34] Broadley M.R., Alcock J., Alford J., Cartwright P., Foot I., Fairweather-Tait S.J., Hart D.J., Hurst R., Knott P., McGrath S.P. (2010). Selenium Biofortification of High-Yielding Winter Wheat (*Triticum aestivum* L.) by Liquid or Granular Se Fertilization. Plant Soil.

[35] Hart D.J., Fairweather-Tait S.J., Broadley M.R., Dickinson S.J., Foot I., Knott P., McGrath S.P., Mowat H., Norman K., Scott P.R. (2011). Selenium Concentration and Speciation in Biofortified Flour and Bread: Retention of Selenium During Grain Biofortification, Processing and Production of Se-Enriched Food. Food Chem..

[36] Coppa E., Celletti S., Sestili F., Mimmo T., Garcia Molina M.D., Cesco S., Astolfi S. (2023). Interaction between Sulfate and Selenate in Tetraploid Wheat (*Triticum turgidum* L.) Genotypes. Int. J. Mol. Sci..

[37] Saeed K., Nisa F.K., Abdalla M.A., Mühling K.H. (2023). The Interplay of Sulfur and Selenium Enabling Variations in Micronutrient Accumulation in Red Spinach. Int. J. Mol. Sci..

[38] Aslam R., Jilani G., Alam T., Haq Z.U., Bhatti A., Ullah R., Naz I., Ikram M., Fatima N., Ali E.A. (2025). Redox Cycling of Sulfur via Microbes in Soil Boosts the Bioavailability of Nutrients to Brassica napus. PLoS ONE.

[39] Rusu M., Mihai M., Tritean N., Mihai V.C., Moldovan L., Ceclan A.O., Russu F., Toader C. (2024). Protection and Modeling in the Use of S, Ca, and Mg Alternatives for Long-Term Sustainable Fertilization Systems. Agronomy.

[40] Guo Q., Ye J., Zeng J., Chen L., Korpelainen H., Li C. (2022). Selenium Species Transforming Along Soil–Plant Continuum and Their Beneficial Roles for Horticultural Crops. Hortic. Res..

[41] Deng G., Fan Z., Wang Z., Peng M. (2025). Dynamic Role of Selenium in Soil–Plant–Microbe Systems: Mechanisms, Biofortification, and Environmental Remediation. Plant Soil.

[42] Bumbadiya N.R., Parmar J.K., Shah S.N., Mehta P.V. (2025). Influence of Iron and Zinc Biofortification on Macro and Micronutrient Dynamics in Durum Wheat Grain and Straw Cultivated in Vertic Ustochrepts Soil. Int. J. Adv. Biochem. Res..

[43] Hu C., Nie Z., Shi H., Peng H., Li G., Liu H., Li C., Liu H. (2023). Selenium Uptake, Translocation, Subcellular Distribution and Speciation in Winter Wheat in Response to Phosphorus Application Combined with Three Types of Selenium Fertilizer. BMC Plant Biol..

[44] Liu H., Shi Z., Li J., Zhao P., Qin S., Nie Z. (2018). The Impact of Phosphorus Supply on Selenium Uptake During Hydroponics Experiment of Winter Wheat (*Triticum aestivum*) in China. Front. Plant Sci..

[45] Roa G.A., Quintana-Obregón E.A., González-Renteria M., Ruiz Diaz D.A. (2024). Increasing Wheat Protein and Yield through Sulfur Fertilization and Its Relationship with Nitrogen. Nitrogen.

[46] Leszczyńska D., Kwiatkowska-Malina J. (2011). Effect of Organic Matter from Various Sources on Yield and Quality of Plants on Soils Contaminated with Heavy Metals. Ecol. Chem. Eng. S.

[47] Ducsay L., Zapletalová A., Slepčan M., Vicianová M., Hozlár P., Bušo R. (2021). Selenium Effect on Wheat Grain Yield and Quality Applied in Different Growth Stages. Plant Soil Environ..

[48] Zembala M., Filek M., Walas S., Mrowiec H., Kornaś A., Miszalski Z., Hartikainen H. (2010). Effect of Selenium on Macro- and Microelement Distribution and Physiological Parameters of Rape and Wheat Seedlings Exposed to Cadmium Stress. Plant Soil.

[49] Brodowska M.S., Filipek T., Kurzyna-Szklarek M. (2017). Content of Magnesium and Calcium in Cultivated Plants Depending on Soil Supply with Nitrogen, Potassium, Magnesium and Sulfur. J. Elem..

[50] Schiavon M., Dall’acqua S., Mietto A., Pilon-Smits E.A.H., Sambo P., Masi A., Malagoli M. (2013). Selenium Fertilization Alters the Chemical Composition and Antioxidant Constituents of Tomato (*Solanum lycopersicon* L.). J. Agri. Food Chem..

[51] Kopsell D.A., Randle W.M., Mills H.A. (2000). Nutrient Accumulation in Leaf Tissue of Rapid-Cycling Brassica Oleracea Responds to Increasing Sodium Selenate Concentrations. J. Plant Nutr..

[52] Hawrylak-Nowak B. (2008). Changes in anthocyanin content as indicator of maize sensitivity to selenium. J. Plant Nutr..

[53] Wu L., Huang Z.Z. (1992). Selenium Assimilation and Nutrient Element Uptake in White Clover and Tall Fescue under the Influence of Sulfate Concentration and Selenium Tolerance of the Plants. J. Exp. Bot..

[54] Brodowska M.S., Kaczor A. (2007). Content of Various Forms of Sulphur in Wheat and Rape Under Conditions of Varied Soil Supply in Sulphur, Magnesium and Calcium. Proc. ECOpole.

[55] Zhang W., He X., Chen X., Han H., Shen B., Diao M., Liu H. (2023). Exogenous Selenium Promotes the Growth of Salt-Stressed Tomato Seedlings by Regulating Ionic Homeostasis, Activation Energy Allocation and CO_2_ Assimilation. Front. Plant Sci..

[56] Yu L., Chen Q., Liao X., Yang X., Chao W., Cong X., Zhang W., Liao Y., Ye J., Xu F. (2023). Exploring Effects of Exogenous Selenium on the Growth and Nutritional Quality of Cabbage (*Brassica oleracea* var. *capitata* L.). Horticulturae.

[57] Li H., Liu X., Wassie M., Chen L. (2020). Selenium Supplementation Alleviates Cadmium-Induced Damages in Tall Fescue Through Modulating Antioxidant System, Photosynthesis Efficiency, and Gene Expression. Environ. Sci. Poll. Res. Int..

[58] Barczak B., Lopuszniak W., Moskal M. (2019). Yield of Spring Barley in Conditions of Sulphur Fertilization. J. Central Europ. Agr..

[59] Winiarska-Mieczan A., Zaricka E., Kwiecień M., Kwiatkowska K., Baranowska-Wójcik E., Danek-Majewska A. (2019). Can Cereal Products Be an Essential Source of Ca, Mg and K in the Deficient Diets of Poles?. Biol. Trace Element Res..

[60] Łukaszewicz S., Politycka B., Smoleń S. (2019). Accumulation of Selected Macronutrients and Tolerance Towards Selenium of garden Pea Treated with Selenite and Selenate. J. Elem..

[61] Hawrylak-Nowak B., Matraszek R., Pogorzelec M. (2015). The Dual Effects of Two Inorganic Selenium Forms on Growth, Selected Physiological Parameters and and Macronutrient Accumulation in Cucumber Plants. Acta Physiol. Plant..

[62] Brodowska M.S., Kurzyna-Szklarek M., Wyszkowski M. (2025). Sulphur and Selenium as Determinants of Yield and Biometric Parameters in Wheat. Agronomy.

[63] Brodowska M.S., Kurzyna-Szklarek M., Wyszkowski M. (2025). Selenium and Sulphur as Elements Modifying Plant Quality: Assessment of the Content of Organic and Mineral Nitrogen Forms in Wheat. Molecules.

[64] Brodowska M.S., Kurzyna-Szklarek M., Wyszkowski M. (2026). Selenium (IV) and Sulphur (VI) as Elements Modifying Plant Quality: Content of Selenium and Sulphur Forms in Wheat. Molecules.

[65] Aspel C., Murphy P.N.C., McLaughlin M.J., Forrestal P.J. (2022). Sulfur Fertilization Strategy Affects Grass Yield, Nitrogen Uptake, and Nitrate Leaching: A Field Lysimeter Study. J. Plant Nutr. Soil Sci..

[66] Kolbe H. (2024). Meta-Study on Sulphur Supply of Various Crop Species in Organic Farming Between 1998 and 2023 in European Countries—Part 2: Effects of S Concentration and N:S Ratio of Different Plant Parts on Dry Biomass, N-Uptake and Legume N_2_ Fixation. Agronomy.

[67] Takahashi H., Marsolais F., Cuypers A., Kopriva S. (2023). Sulfur Metabolism: Actions for Plant Resilience and Environmental Adaptation. J. Experim. Bot..

[68] Abdalla M.A., Sulieman S., Mühling K.H. (2020). Regulation of Selenium/Sulfur Interactions to Enhance Chemopreventive Effects: Lessons to Learn from Brassicaceae. Molecules.

[69] Silva V.M., Wilson L., Young S.D., Broadley M.R., White P.J., Reis A.R. (2023). Interaction Between Sulfur and Selenium in Agronomic Biofortification of Cowpea Plants Under Field Conditions. Plant Soil.

[70] IUSS Working Group WRB (2015). World Reference Base for Soil Resources 2014; International Soil Classification System for Naming Soils and Creating Legends for Soil Maps. World Soil Resources Reports No. 106.

[71] Ostrowska A., Gawliński S., Szczubiałka Z. (1991). Methods for Analysis and Evaluation of Soil and Plant Properties.

[72] TIBCO Software Inc. (2017). Statistica (Data Analysis Software System).

[73] Gurvich V., Naumova M. (2021). Logical Contradictions in the One-Way ANOVA and Tukey–Kramer Multiple Comparisons Tests with More Than Two Groups of Observations. Symmetry.

